# Endophytic Fungi: A Source of Potential Antifungal Compounds

**DOI:** 10.3390/jof4030077

**Published:** 2018-06-25

**Authors:** Sunil K. Deshmukh, Manish K. Gupta, Ved Prakash, Sanjai Saxena

**Affiliations:** 1 TERI-Deakin Nano Biotechnology Centre, The Energy and Resources Institute (TERI), Darbari Seth Block, IHC Complex, Lodhi Road, New Delhi 110003, India; manish.gupta@teri.res.in; 2 Department of Biotechnology, Motilal Nehru National Institute of Technology, Allahabad 211004, India; ved.prakash2012@vitalum.ac.in; 3 Department of Biotechnology, Thapar Institute of Engineering & Technology, Deemed to be a University, Patiala, Punjab 147004, India; sanjaibiotech@yahoo.com

**Keywords:** endophytic fungi, antifungal compounds, medicinal plants, co-culture, epigenetic modification

## Abstract

The emerging and reemerging forms of fungal infections encountered in the course of allogeneic bone marrow transplantations, cancer therapy, and organ transplants have necessitated the discovery of antifungal compounds with enhanced efficacy and better compatibility. A very limited number of antifungal compounds are in practice against the various forms of topical and systemic fungal infections. The trends of new antifungals being introduced into the market have remained insignificant while resistance towards the introduced drug has apparently increased, specifically in patients undergoing long-term treatment. Considering the immense potential of natural microbial products for the isolation and screening of novel antibiotics for different pharmaceutical applications as an alternative source has remained largely unexplored. Endophytes are one such microbial community that resides inside all plants without showing any symptoms with the promise of producing diverse bioactive molecules and novel metabolites which have application in medicine, agriculture, and industrial set ups. This review substantially covers the antifungal compounds, including volatile organic compounds, isolated from fungal endophytes of medicinal plants during 2013–2018. Some of the methods for the activation of silent biosynthetic genes are also covered. As such, the compounds described here possess diverse configurations which can be a step towards the development of new antifungal agents directly or precursor molecules after the required modification.

## 1. Introduction

The undisputed potential of fungi to produce bioactive secondary metabolites has long been established. To date, merely 5% of the entire number of species has been elaborated of an estimated 1.5 million. Out of these (69,000 species), merely 16% (11,500) have been cultured [1]. Amongst the fungi, endophytes represent a wide source of unexplored and uncharacterized microorganisms capable of producing novel metabolites. Endophytes generally exist asymptomatically, coexisting with their hosts and representing an underutilized group of microorganisms for the discovery of new compounds. Endophytes produce diverse metabolites and have the ability to synthesize compounds which are solely produced and isolated from higher plants [2,3]. Strobel and Daisy [4] commented that endophytes could be a goldmine of secondary metabolites. *Pestalotiopsis* sp. can be considered as “the *E. coli* of the rain forests” and *P. microspora*, a “microbial factory” of bioactive secondary metabolites. As per them, numerous chemical structures such as Ambuic acid, Cryptocandin, Taxol, Torreyanic acid, Subglutinol A and B, and many others have been identified. An array of metabolites of different chemical classes profiles have been deciphered, such as alkaloids, cytochalasines, flavonoids, furandiones, phenylpropanoids, lignans, peptides, phenol, phenolic acids, steroids, terpenoids, quinones, aliphatic acid, and chlorinated compounds. Secondary metabolites derived from endophytes comprise classes of compounds such as steroids, xanthones, phenols, isocoumarins, perylene derivatives, quinines, furandiones, terpenoids, depsipeptides, and cytochalasins, which are identified to possess biological activities with antibiotic, antiviral, volatile antibiotic, anticancer, antioxidant, insecticidal, antidiabetic, and immunosuppressive properties [5,6,7,8,9]. Endophytes play a major role in the physiological activities of host plants, influencing the enhancement of stress, insects, nematodes, and disease resistance [10,11,12,13].

This review covers the antifungal fungal metabolites reported from endophytic fungi from medicinal plants during 2013–2018 and their potential as antifungal agents. The antifungal activity of these compounds against the selected fungal pathogens are described briefly and some details such as producing organisms, plant sources, place of collections, and the antifungal properties of many of these compounds are shown in Table 1 and Table 2.

## 2. Medicinal Plants

### 2.1. Compounds Produced by Coelomycetes

**Figure 1:**
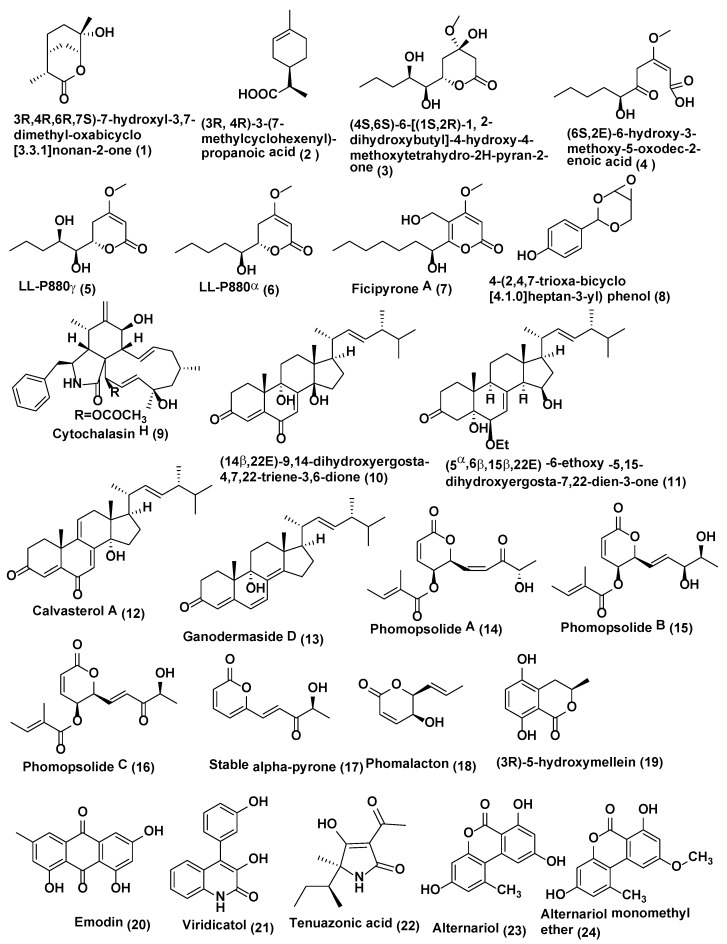
Structures of metabolites isolated from Coelomycetes (**1**–**24**).

*Pestalotiopsis* is an important genus of coelomycetes and different species of this genus have been identified for production of bioactive compounds for various biological properties which include antimicrobial, antifungal, antiviral, antineoplastic, and antioxidant activities [14]. Some of the antifungal reported from this genus includes a new monoterpene lactone, (3R,4R,6R,7S)-7-hydroxyl-3,7-dimethyl-oxabicyclo[3.3.1]nonan-2-one (**1**) (Figure 1), along with one related known compound, (3R,4R)-3-(7-methylcyclohexenyl)-propanoic acid (**2**) (Figure 1), were discovered from endophytic fungus *Pestalotiopsis foedan* obtained from the branch of *Bruguiera sexangula* in Hainan, China. Compounds **1**–**2** showed antifungal activity against *Botrytis cinerea* and *Phytophthora nicotianae* with MIC values of 3.1 and 6.3 µg/mL, respectively, while the known antifungal drug ketoconazole showed comparable activity (MIC 3.1 µg/mL each) Compound **2** also exhibited satisfactory activity against *Candida albicans* (MIC value of 50 µg/mL) while ketoconazole showed MIC of 6.3 µg/mL [15].

Plants of *Dendrobium officinale* were collected in Yandang Mountain, Zhejiang Province, China. *Pestalotiopsis* sp. DO14 was obtained from the shoots of *D. officinale* endophytic fungus which yielded two novel antifungal constituents, (4S,6S)-6-[(1S,2R)-1,2-dihydroxybutyl]-4-hydroxy-4-methoxytetrahydro-2H-pyran-2-one (**3**) and (6S,2E)-6-hydroxy-3-methoxy-5-oxodec -2-enoic acid (**4**), and two known compounds, LL-P880γ (**5**) and LL-P880α (**6**) (Figure 1) were isolated. Compounds **3**–**6** exhibited good anti-fungal activities (MIC ≤ 50 µg/mL) against *C. albicans*, *Cryptococcus neoformans*, *Trichophyton rubrum*, and *Aspergillus fumigatus.* Compounds **3** and **4** possess the strong activities with the MIC values ≤25 µg/mL against tested strains [16].

Endophytic fungus *Pestalotiopsis fici* obtained from the branches of *Camellia sinensis* collected from the suburb of Hangzhou, China was the source of a new a-pyrone derivative ficipyrone A (**7**) (Figure 1). Compound **7** showed antifungal activity against the plant pathogen *Gibberella zeae* with an IC_50_ value of 15.9 µM (the positive control ketoconazole showed an IC_50_ value of 6.02 µM) [17].

Endophytic fungus *Pestalotiopsis mangiferae* associated with *Mangifera indica* Linn collected from Maduravoyal, Tamil Nadu Province, India was the source of a new phenolic compound 4-(2,4,7-trioxa-bicyclo[4.1.0]heptan-3-yl) phenol (**8**) (Figure 1). Compound **8** exhibited strong antifungal activity against *C. albicans* with MIC value of 0.039 µg/mL, while nystatin showed MIC 10.0 µg/mL [18].

*Phomopsis* is an important genus that is a prolific producer of bioactive compounds including Cytochalasin H (**9**) (Figure 1), which was isolated from the endophytic fungus *Phomopsis* sp. of *Senna spectabilis* (Fabaceae) collected from São Paulo, Brazil. Compound **9** exhibited activity against *Cladosporium cladosporioides* and *C. sphaerosphermum* with MIC values of 10.0 and 25.0 µg, respectively, while the MIC of nystatin the reference compound was 1.0 µg [19].

Endophytic fungus *Phomopsis* sp. isolated from *Aconitum carmichaeli* collected in Huize County, Yunnan Province, China yielded (14β,22E)-9,14-dihydroxyergosta-4,7,22-triene-3,6-dione (**10**) and (5α,6β,15β,22E)-6-ethoxy-5,15 -dihydroxyergosta-7,22-dien-3-one (**11**), calvasterol A (**12**), and ganodermaside D (**13**) (Figure 1). All compounds were evaluated for their antifungal activities against *Candida albicans*, *Aspergillus niger*, *Pyricularia oryzae*, *Fusarium avenaceum*, *Hormodendrum compactum*, and *Trichophyton gypseum.* Compound **10** exhibited average antifungal activities against *C. albicans*, *H. compactum*, and *A. niger*, with MIC values of 64, 64, and 128 µg/mL, respectively. Compound **11** showed poor inhibitory activity against *C. albicans* and *F. avenaceum* with MIC values of 128 µg/mL. Compounds **12** and **13** showed average inhibitory activities against *F. avenaceum* (MIC 64 µg/mL for both compounds). Only compound **12** exhibited weak antifungal activities against *P. oryzae* and *T. gypseum* (MIC of 128 and 256 µg/mL, respectively) [20].

*Diaporthe maritima* an endophytic fungus obtained from needles of *Picea* sp. in the Acadian forest of Eastern Canada yielded three dihydropyrones, phomopsolides A (**14**), B (**15**), and C (**16**), and a stable alpha-pyrone (**17**) (Figure 1). Compound **14** demonstrated growth inhibition at 25 µM against *Microbotryum violaceum* and *Saccharomyces cerevisiae* whereas Compounds **15**–**17** were active at 250 µM [21].

Another coelomycete *Phoma* is known to produce diverse compounds [22]. From *Phoma* sp. an endophytic fungus of the plant *Fucus serratus* yielded phomalacton (**18**), (3R)-5-hydroxymellein (**19**) and emodin (**20**) (Figure 1). Phomalactone (**18**), (3R)-5-hydroxymellein (**19**) and emodin (**20**) Compounds **18**–**20** exhibited antifungal activity against *Microbotryum violaceum* with 5, 6 and 5 mm zone of inhibition (0.05 mg was pipetted onto 9 mm sterile filter disk) [23].

Viridicatol (**21**) (Figure 1), tenuazonic acid (**22**), alternariol (**23**), and alternariol monomethyl ether (**24**) (Figure 1) were isolated from endophytic fungi *Phoma* sp. WF4 of *Eleusine coracana* grown under semi-hydroponic conditions Arkell Field Station, Arkell, ON, Canada. Compounds **21**–**24** caused reasonable breakage of *Fusarium graminearum* hyphae in vitro [24].

**Figure 2:**
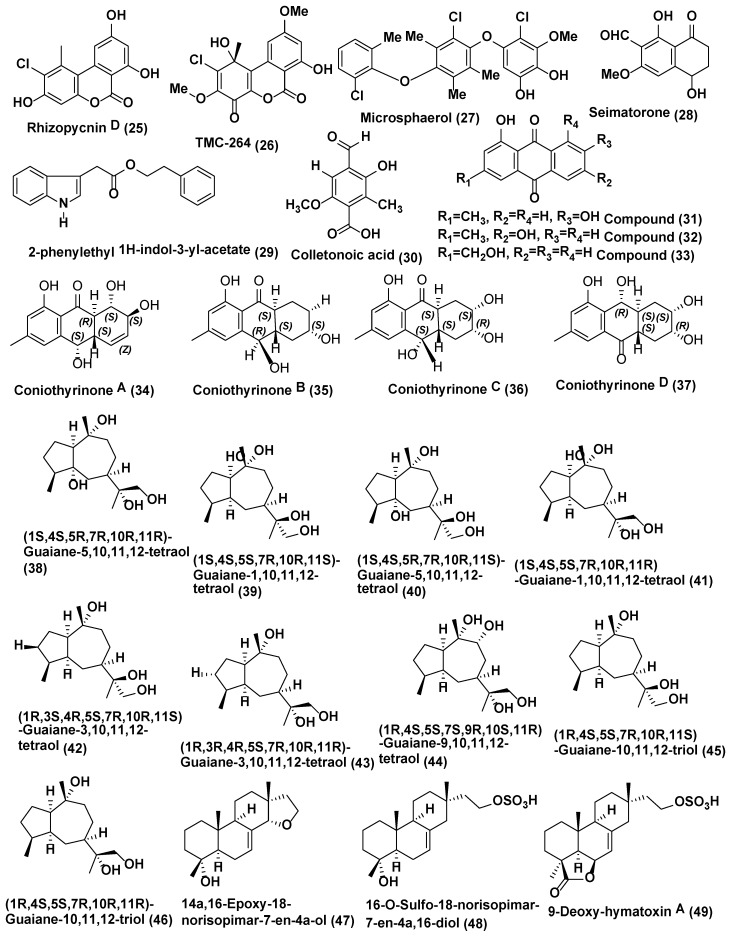
Structures of metabolites isolated from Coelomycetes (**25**–**37**) and Ascomycetes (**38**–**49**).

Endophytic fungus *Rhizopycnis vagum* Nitaf 22 obtained from the healthy root of *Nicotiana tabacum* grown at China Agricultural University Beijing, China was the source of Rhizopycnin D (**25**) and TMC-264 (**26**) (Figure 2). Compounds **25** and **26** showed strong inhibition of the spore germination of *Magnaporthe oryzae* with IC_50_ values of 9.9 and 12.0 µg/mL, respectively [25].

A new polychlorinated triphenyl diether named microsphaerol (**27**) (Figure 2) has been isolated from the endophytic fungus *Microsphaeropsis* sp. (internal strain No. 8883), and phytochemical investigation of the endophytic fungus *Seimatosporium* sp. (internal strain No. 8883) associated with *Salsola oppositifolia* from Playa del Ingles (Gomera, Spain) led to the isolation of a new naphthalene derivative named seimatorone (**28**) (Figure 2). In antifungal assay, compounds **27** and **28** showed activity against *Microbotryum violaceum* with 9 and 5 mm zone of inhibition (0.05 mg was pipetted onto 9 mm sterile filter paper disk). In addition, there was some growth within the zone of inhibition [26].

Endophytic fungus *Colletotrichum gloeosporioides* associated with *Michelia champaca* isolated from São Paulo State University (UNESP), Araraquara, São Paulo, Brazil was found to be source of a new compound, 2-phenylethyl 1H-indol-3-yl-acetate (**29**) (Figure 2). Compound **29** displayed good activity against *Cladosporium cladosporioides* and *C. sphaerospermum* which was analogous to nystatin, the positive control [27].

Colletonoic acid (**30**) (Figure 2) was isolated from *Colletotrichum* sp. from Gomera (Spain). Colletonoic acid exhibit antifungal activity against *Microbotryum violaceum* with 7 mm zone of inhibition (0.05 mg was pipetted onto 9 mm a sterile filter paper disk) [28].

*Coniothyrium* sp., an endophytic fungus associated with *Salsola oppostifolia* from Gomera in the Canary Islands, was the source of known hydroxy anthraquinones 1,7-dihydroxy3-methyl-9,10-anthraquinone (**31**), 1,6-dihydroxy-3-methyl-9,10-anthraquinone (phomarin) (**32**), and 1-hydroxy-3-hydroxymethyl-9,10-anthraquinone (**33**) (Figure 2) along with four new derivatives having a tetralone moiety, namely coniothyrinones A–C (**34**–**36**) (Figure 6) and D (**37**) (Figure 2). The absolute configurations of coniothyrinones A (**34**), B (**35**), and D (**37**) were determined by TDDFT calculations of CD spectra, allowing the determination of the absolute configuration of coniothyrinone C (**36**) as well. Coniothyrinones A (**34**), B (**35**), and D (**37**) could be used as ECD reference compounds in the determination of absolute configuration for related tetralone derivates. Compounds **31**–**37** showed inhibitory effects against the fungus *Microbotryum violaceum* with 7, 10, 8, 7.5, 6, 8 and 7.5 mm zone of inhibition (0.05 mg/9-mm sterile filter paper disk). Compounds **32** and **34** exhibited strong antifungal activity against *M. violaceum* (10 and 9 mm zone of inhibition) and *B. cinerea* (7.5 and 12.5 mm zone of inhibition) when tested under similar conditions [29].

### 2.2. Compounds Produced by Ascomycetes

*Xylaria* is very important ascomycetous genus and a good sources of novel bioactive compounds, and some of the compounds reported to have drug-able properties relevant for drug discovery [30,31]. Nine oxygenated guaiane-type sesquiterpenes (**38**–**46**) and three isopimarane diterpenes (**47**–**49**), (Figure 2) were obtained from *Xylaria* sp. YM 311647, an endophytic fungus associated with *Azadirachta indica* collected from Yuanjiang County, Yunnan Province, China. All compounds were evaluated for their antifungal activities against *Candida albicans*, *Aspergillus niger*, *Pyricularia oryzae*, *Fusarium avenaceum*, and *Hormodendrum compactum*. Compounds **38**–**46** were moderately active against *C. albicans* and *H. compactum* (MIC values ranging from 32 to 256 µg/mL), while compound **47**–**49** were more active against all the tested strains (MIC values ranging from 16 to 256 µg/mL). Compound **49** exhibited the most promising activity against *C. albicans* and *P. oryzae* with MIC values of 16 µg/mL [32].

**Figure 3:**
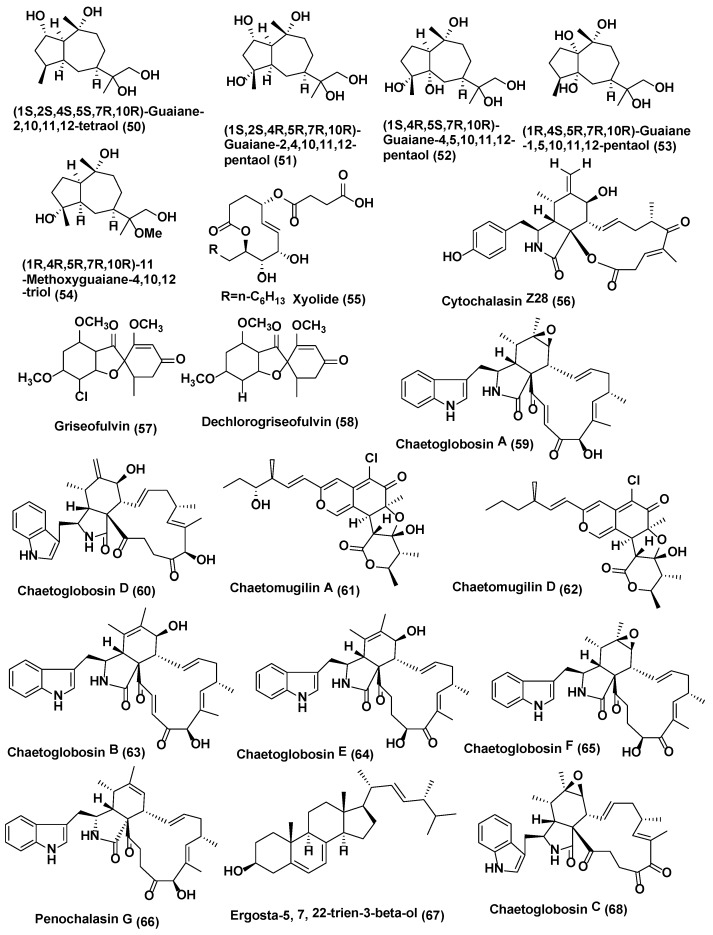
Structures of metabolites isolated from Ascomycetes (**50**–**68**).

Endophytic fungus *Xylaria* sp. YM 311647 associated with *Azadirachta indica* from Yuanjiang County, China was also reported to produce five new guaiane sesquiterpenes, (**50**–**54**) (Figure 3). The antifungal activities of **50**–**54** were evaluated by means of the broth microdilution method against *C. albicans*, *A. niger*, *P. oryzae*, *F. avenaceum* and *H. compactum* Compounds **50**–**54** exhibited average or poor antifungal activities against *P. oryzae* and *H. compactum* (MIC values in the range of 32–256 µg/mL). Among them, **53** exhibited the most promising inhibitory activity against *P. oryzae* with a MIC value of 32 µg/mL. Compounds **52** and **53** showed average antifungal activities against *H. compactum* with MIC values of 64 µg/mL. In addition, **53** and **54** exhibited the most promising antifungal activities against *C. albicans* with MIC values of 32 µg/mL. Compound **52** showed average inhibitory activities against *C. albicans*, *A. niger*, and *H. compactum* with MIC values of 64 µg/mL. All compounds showed no notable inhibitory activities against *Fusarium avenaceum* [33]. Amazonian endophytic fungus *X. feejeensis* residing in *Croton lechleri* yielded nonenolide, xyolide (**55**) (Figure 3). Compound **55** exhibited antifungal activity against oomycetes *Pythium ultimum* with a MIC value of 425 µM [34].

Endophytic fungus *Xylaria sp.* XC-16 associated with *Toona sinensis* was isolated from Yangling, Shaanxi Province, China and was observed to produce a potent antifungal compound Cytochalasin Z28 (**56**) (Figure 3), displaying enhanced activity with an MIC of 12.5 µM as opposed to the antifungal activity possessed by hymeaxszol possessing an MIC value of 25 µM against the plant pathogen *Gibberella saubinetti* [35].

Various isolates of *Xylaria* produce griseofulvin (**57**) (Figure 3); *Xylaria sp.* PSU G12 associated with *Garcinia hombroniana* [36], and *X. cubiensis* residing in *Asimina triloba* [37], along with 13 strains of *Xylaria* sp. inhabiting *Pinus strobus* and six strains associated with *Vaccinum augustifolium* found in the Acadian forest of New Brunswick and Nova Scotia Canada [38] are known to produce griseofulvin and a few of them can also produce dechlorogriseofulvin (**58**) (Figure 3) [36,38].

Griseofulvin (**57**) is very potent against the phytopathogenic fungi, but not against oomycetes [38]. Griseofulvin has been validated to possess antifungal activity against *Alternaria mali*, *B. cinerea*, *C. gloeosporioides*, *Corticium sasaki*, *Fusarium oxysporum* and *Magnaporthe grisea* in vitro with IC_50_ values of 18.0, 5.0, 1.7, 11.0, 30.0, and 1.7 µg/mL, respectively. Dechlorogriseofulvin (**58**) demonstrated poor activity, with an IC_50_ value of 200 µg/mL for each fungus. Griseofulvin (**57**) also hinders the growth of *M. grisea*, *C. sasaki*, *B. cinerea*, *Puccinia recondite* and *Blumeria graminis* f. sp. *hordei* in vivo, with a percentage of fungal control of 95, 100, 60, 90 and 90, respectively, at 150 µg/mL. Griseofulvin (**57**) is used to cure dermatophytic infections caused by fungi such as *Epidermophyton* and *Trichophyton* species [38].

*Chaetomium* is another genus of ascomycete and prolific producer of bioactive compounds [39,40]. Chaetoglobosin A (**59**) and D (**60**) (Figure 3) were isolated *Chaetomium globosum* CDW7, an endophyte from *Ginkgo biloba* located in Taixing and Nanjing in Jiangsu Province and Chengdu in Sichuan Province, China. Compounds **59** and **60** showed antifungal activity against *Sclerotinia sclerotiorum* with IC_50_ values of 0.35 and 0.62 µg/mL, respectively, compared with carbendazim (0.17 µg/mL) [41].

Compounds Chaetomugilin A (**61**)**,** Chaetomugilin D (**62**) Chaetoglobosin A (**59**), Chaetoglobosin B (**63**), Chaetoglobosin E (**64**), Chaetoglobosin F (**65**), and Penochalasin G (**66**) (Figure 3) were obtained from *C. globosum* endophyte obtained from seeds of *Panax notoginseng* collected at the Wenshan, Yunnan, China. Compounds **59** and **61**–**66** exhibited antifungal activity against *Phoma herbarum* (MIC in the range of 16–128 µg/mL) and *Epicoccum nigrum* (MIC in the range of <1–16 µg/mL). Both fungi are phytopathogenic fungi causing root rot of *Panax notoginseng* [42].

Ergosta-5,7,22-trien-3beta-ol (**67**) (Figure 3) was isolated from *Chaetomium cupreum* ZJWCF079 of *Macleaya cordata.* It exhibited antifungal activity against *Sclerotinia sclerotiorum* and *B. cinereal*, plant pathogenic fungi with EC_50_ values of 125 µg/mL and 190 µg/mL respectively, but had no effects on *Pythium ultimum*, *Rhizoctonia solani* and *F. oxysporum* [43].

**Figure 4:**
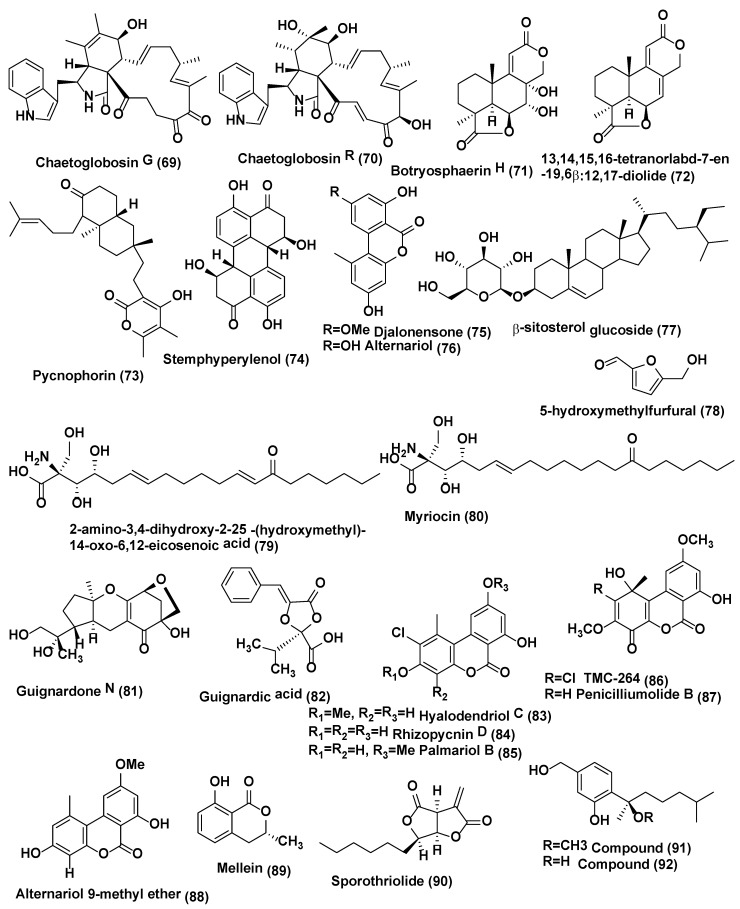
Structures of metabolites isolated from Ascomycetes (**69**–**92**).

Chaetoglobosin A (**59**) (Figure 3), D (**60**), E (**64**), C (**68**), (Figure 3) G (**69**), and R (**70**) (Figure 4) were isolated from *Chaetomium globosum* No.04 obtained from barks of *Ginkgo biloba*, growing in Linyi, Shandong Province, China. Compounds **59**, **60**, **64**, and **68**–**70** showed good growth inhibitory activity at a concentration of 20 µg/disk, against *Rhizopus stolonifer* and *Coniothyrium diplodiella* [44].

A new tetranorlabdane diterpenoids botryosphaerin H (**71**) and a known tetranorlabdane diterpenes 13,14,15,16-tetranorlabd-7-en-19,6β:12,17-diolide (**72**) (Figure 4) were obtained from *Botryosphaeria* sp. P483, an endophyte of *Huperzia serrata* collected in Xichou County, Yunnan Province, China. When tested at 100 µg/disk, compound **71** showed zone of inhibition of 9, 7, 7, 8, and 8 mm, against *Gaeumannomyces graminis*, *Fusarium solani*, *Pyricularia oryzae*, *Fusarium moniliforme*, and *F. oxysporum* while compound **72** showed zone of inhibition of 12, 10, 10, 11, 13 mm against *G. graminis*, *F. solani*, *P. oryzae*, *F. moniliforme*, and *F. oxysporum.* The standard Carbendazim (50 µg/disk) exhibited activity against *G. graminis*, *F. solani*, *P. oryzae*, *F. moniliforme*, and *F. oxysporum*, with the zone of inhibition of 14, 18, 15, 17 and 15, mm respectively [45].

Endophytic fungus *Botryosphaeria dothidea* KJ-1 associated with the stems of *Melia azedarach* collected at Yangling, Shaanxi Province, China was the source of pycnophorin (**73**), stemphyperylenol (**74**), chaetoglobosin C (**68**), djalonensone (**75**) (Figure 4), alternariol (**76**), β-sitosterol glucoside (**77**), and 5-hydroxymethylfurfural (**78**) (Figure 4). Stemphyperylenol (**74**) exhibited good antifungal activity against *Alternaria solani* the plant pathogen with the MIC value of 1.57 µM comparable to commonly used fungicide, the carbendazim. Compounds **68**, **73**, and **75**–**78** showed good to average antifungal activities against *A. solani* (MICs of 6.25−25 µM) [46].

Two eicosanoic acids, 2-amino-3,4-dihydroxy-2-25-(hydroxymethyl)-14-oxo-6,12-eicosenoic acid (**79**) and myriocin (**80**) (Figure 4), were isolated from *Mycosphaerella* sp. an endophytic fungus of *Eugenia bimarginata* DC. (Myrtaceae) collected in Brazil (Savannah). These compounds displayed antifungal activities against several isolates of *C. neoformans* and *C*. *gattii*, with MIC values for compound **79** ranging from 1.3 to 2.50 µg/mL and for compound **80** was 0.5 µg/mL [47]. Both compounds exhibited antifungal activities against several isolates of *C*. *neoformans* and *C. gattii*, with MIC values ranging from 0.49 to 7.82 µM for compound **79** and 0.48–1.95 µM for compound **80** in another study. When checked by the checkerboard microtiter assay, both compounds exhibited synergistic activity against *C. gattii* with amphotericin B. Ultrastructural analysis divulges various signs of damage in *C. gattii* and *C. neoformans* cells treated with compounds **79** and **80**, including deformities in cell shape, depressions on the surface, and withered cells. Compounds **79** and **80** showed less loss of cellular material in cells of *C. gattii* compared to those treated with amphotericin B.

The difference in cellular material loss increased in a test compound concentration-dependent manner. Compound **80** also induced the formation of several pseudohyphae, suggesting that it could reduce virulence in *C. gattii* cells [48].

Endophytic fungus *Guignardia* sp., associated with *Euphorbia sieboldiana* collected from Nanjing, Jiangsu, China was the source of guignardone N (**81**) and guignardic acid (**82**) (Figure 4). Both compounds were evaluated for their inhibitory effects alone and with fluconazole on the growth and biofilms of *Candida albicans*. At 6.3 µg/mL combined with 0.031 µg/mL of fluconazole, compounds **81** and **82** were found to have prominent inhibition on the growth of *C. albicans* with fractional inhibitory concentration (FIC) index values of 0.23 and 0.19, respectively. Combined with fluconazole, both (40 µg/mL for (**81**) and 20 µg/mL for (**82**) could also inhibit *C. albicans* biofilms and reverse the tolerance of *C. albicans* biofilms to fluconazole [49].

Antifungal hyalodendriol C (**83**), rhizopycnin D (**84**), palmariol B (**85**), TMC-264 (**86**), penicilliumolide B (**87**) and alternariol 9-methyl ether (**88**) (Figure 4) were obtained from the endophytic fungus *Hyalodendriella* sp. Ponipodef 12 associated with the healthy stems of the “Neva” hybrid of *Populus deltoides* Marsh × *P. nigra* L. were collected from Longhua in Hebei Province of China. Compound **83** displayed antifungal effects against the spore germination of *M. oryzae* with potent inhibition with the IC_50_ value of 11.6 µg/mL, which was comparable with the positive control, carbendazim (IC_50_ 6.9 µg/mL) [50]. Previously, it found that rhizopycnin D (**84**), palmariol B (**85**), TMC-264 (**86**), penicilliumolide B (**87**), and alternariol 9-methyl ether (**88**) exhibits antifungal activity against the spore germination of *M. oryzae* [51,52].

Mellein (**89**) (Figure 4), was isolated from *Pezicula* sp. associated with the twigs of *Forsythia viridissima*, Zhejiang Province, Southeast China. Antifungal activity of this compound was tested against *B. cinerea*, *Pythium ultimum*, *Fusarium oxysporium* f. sp. *cucumerinum*, *Colletotrichum orbiculare*, *Verticillium dahliae*, *Pyricularia oryzae*, *Pestalotia diospyri*, *Sclerotinia sclerotiorum* and *Fulvia fulva*. Compound **89** displayed antifungal activity against 9 plant pathogenic fungi, esp. *B. cinerea* and *F. fulva* with EC_50_ values below 50 µg/mL [53].

Endophytic fungus *Nodulisporium* sp. A21 associated with the leaves of *Ginkgo biloba* collected from Nanjing in Jiangsu Province, China was a source of anti-phytopathogenic sporothriolide (**90**) (Figure 4). In mycelia growth inhibition method, sporothriolide (**90**) showed antifungal activity against *Rhizoctonia solani* with the EC_50_ value of 3.04 µg/mL (11.6 µM) while the EC_50_ of positive control carbendazim was 1.84 µg/mL (9.6 µM). Sporothriolide (**90**) at 200 µg/mL had a protective efficacy of 71.7% against Rice Sheath Blight in comparison with the protective efficacy 90.1% of the positive control of validamycin A at 200 µg/mL. Conidia of *Magnaporthe oryzae* could not form the germ tube and appressorium germinate in the sporothriolide with solution at the concentration of 1.5 µg/mL (5% DMSO). In vivo, sporothriolide at 50 µg/mL, compared to tricyclazole of 2.5 µg/mL, could control the developing of Rice Blast [54]. Sporothriolide (**90**) also showed antifungal activity against *Sclerotinia sclerotiorum* with EC_50_ of sporothriolide against was 2.78 µg/mL (10.7 µM) while the EC_50_ of positive control carbendazim was 0.17 µg/mL (0.89 µM). When it comes to the protective activity on rape leaves, the positive control carbendazim of 250 µg/mL was 57.6% and sporothriolide of 250 µg/mL was 41.5% [54].

**Figure 5:**
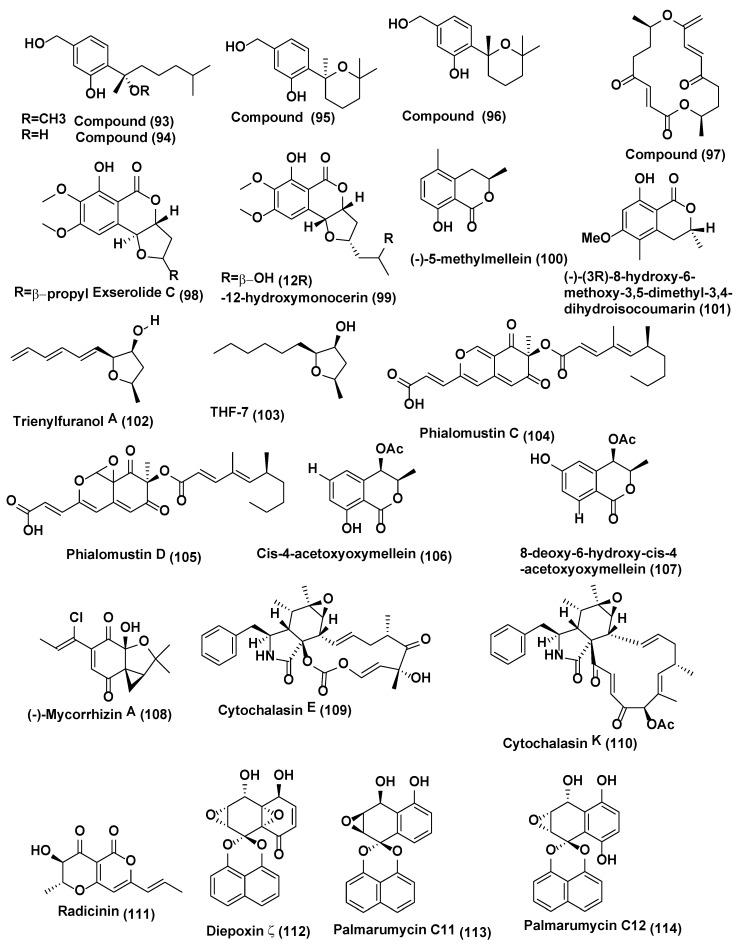
Structures of metabolites isolated from Ascomycetes (**93**–**114**).

Six phenolic bisabolane-type sesquiterpenoids (**91**, **92**) (Figure 4) (**93**–**96**), along with a macrolide, pyrenophorin (**97**) (Figure 5) were isolated from *Lopherdermium nitens* DAOM 250027 endophyte of *Pinus strobus* (eastern white pine) near Sussex, NB, Canada. These compounds were characterized based on interpretation of spectroscopic data (NMR, OR, UV) and HRMS. All compounds were tested for antifungal activity. Pyrenophorin (**97**) significantly reduced the growth of *Microbotryum violaceum* and *Saccharomyces cerevisiae* at 5 µM, whereas sesquiterpenoids (**91**–**96**) were antifungal at 50 µM to both species tested [55]. Isocoumarin derivative exserolide C (**98**) and (12R)-12-hydroxymonocerin (**99**) (Figure 5) were isolated from endophyte *Exserohilum* sp. associated with *Acer truncatum* collected from Beijing, China. Compounds **98** and **99** displayed antifungal activity against *Fusarium oxysporum*, with MIC value of 20 µg/mL for both compounds, while Amphotericin B the positive control showed the MIC value of 0.63 µg/mL [56].

Endophytic fungus of *Echinacea purpurea* associated with *Biscogniauxia mediterranea* EPU38CA from the wild in Missouri, USA was the source of (−)-5-methylmellein (**100**) and (−)-(3R)-8-hydroxy-6-methoxy-3,5-dimethyl-3, 4-dihydroisocoumarin (**101**) (Figure 5). Compound **100** exhibited poor activity against *Phomopsis obscurans*, *P. viticola*, and *Fusarium oxysporum*, and stimulated the growth of *Colletotrichum fragariae*, *C. acutatum*, *C. gloeosporioides*, and *B. cinerea*. Compound **101** was acknowledged to be marginally more active in the microtiter method than 5-methylmellein [57]. Compound **101** was found to be slightly more active in the microtiter method than 5-methylmellein [57]. Trienylfuranol A (**102**) (Figure 5) was isolated from isolated from *Hypoxylon submonticulosum* the endophyte of *Rubus idaeus* collected from Jordan Station, ON, Canada. It was identified based on high-resolution LC-MS and 1- and 2-D NMR spectroscopy. Absolute stereochemical configurations of the compounds were confirmed by NOE NMR experiments and by the preparation of Mosher esters. Complete hydrogenation of I yielded THF 7 (**103**) (Figure 5) that was used for stereochemical characterization and assessment of antifungal activity. Compound THF 7 (**103**) significantly inhibited the growth of *Saccharomyces cerevisiae* (74 ± 4% inhibition) at a concentration of 250 µg/mL as compared with complete inhibition by nystatin at 10 µg/mL [58].

Endophytic fungus *Phialophora mustea* associated with *Crocus sativus* was the source of an unprecedented azaphilone derived skeleton, Phialomustin C (**104**) and D (**105**) (Figure 5). Compounds **104** and **105** showed potent activities against *Candida albicans*, with IC_50_ values of 14.3 and 73.6 µM respectively [59].

**Figure 6:**
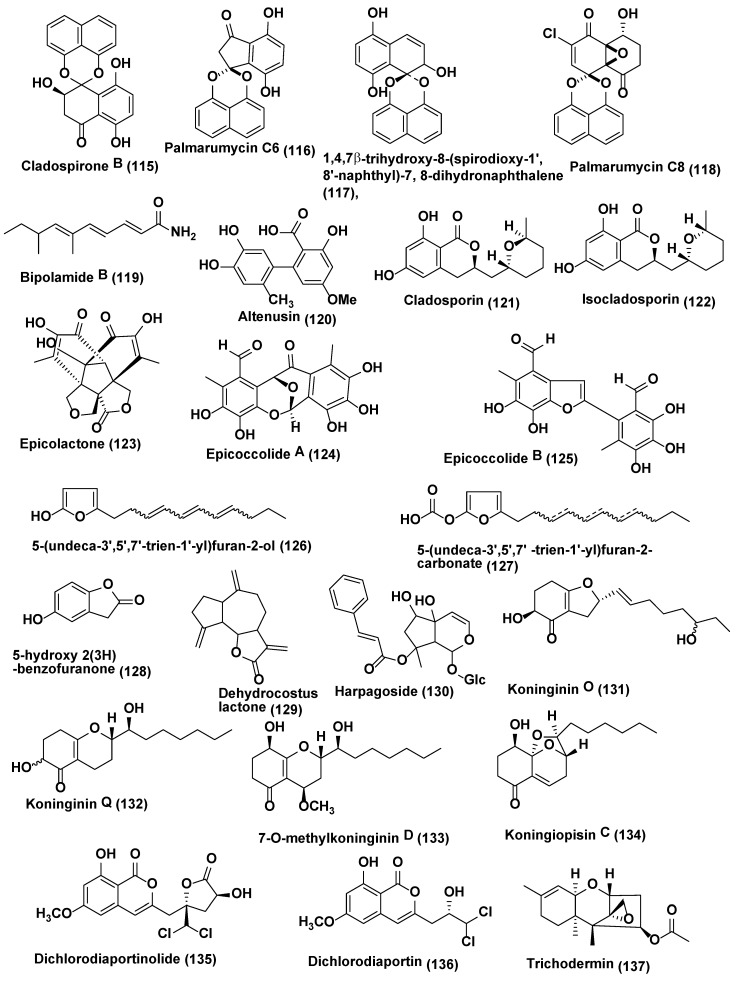
Structures of metabolites isolated from Ascomycetes (**115**–**127**) and Hyphomycetes (**128**–**137**).

An unidentified ascomycete, associated with *Melilotus dentatus* was the source of two new polyketide metabolites *cis*-4-acetoxyoxymellein (**106**) and 8-deoxy-6-hydroxy-*cis*-4-acetoxyoxymellein (**107**) (Figure 5). Compounds **106** and **107** displayed potent antifungal activities toward *Microbotryum violaceum* and *B. cinerea*, with 8 mm zone of inhibition for both fungi tested (0.05 mg was pipetted onto 9 mm sterile filter paper disk). In the case of *B. cinereal*, there was some growth within the zone of inhibition [60].

(−)-Mycorrhizin A (**108**) (Figure 6) was isolated from *Plectophomella* sp. while cytochalasins E (**109**) and K (**110**) (Figure 5) were isolated from *Physalospora* sp. Similarly, radicinin (**111**) (Figure 5) was purified from the endophytic fungus *Crataegus monogyna.* (-)-Mycorrhizin A showed good antifungal activity towards *Ustilago violacea* and *Eurotium repens*. Cytochalasins E (**109**) and K (**110**) showed potent activity against *E. repens* and *Mycotypha microspora*. Radicinin (**111**) (Figure 5) showed good activity against *E. repens* and *M. microspore* [61].

Diepoxin ζ (**112**), palmarumycin C11 (**113**), palmarumycin C12 (**114**) (Figure 5), cladospirone B (**115**), palmarumycin C6 (**116**), 1,4,7β-trihydroxy-8-(spirodioxy-1′,8′-naphthyl)-7,8-dihydronaphthalene (**117**), and palmarumycin C8 (**118**) (Figure 6) were obtained from *Berkleasmium* sp., an endophyte associated with *Dioscorea zingiberensis* from Hubei Province, China. Compounds **112**–**118** were evaluated for their antifungal activity against the spore germination of *M. oryzae*. Compounds **112**–**118** inhibited spore germination of *M. oryzae* with IC_50_ values in the range 9.1–124.5 µg/mL. Palmarumycin C8 (**118**) showed the best inhibitory activity (IC_50_ 9.1 µg/mL) among the compounds tested, although not as active as the positive control carbendazim (IC_50_ 6.3 µg/mL) [62].

Bipolamide B (**119**) (Figure 6) was isolated from *Bipolaris* sp. MU34, the endophytic fungus associated with the leaves of *Gynura hispida* Thwaites collected from Mahidol University, Bangkok, Thailand. The compounds were characterized based on NMR and MS experiments. Bipolamide B (**119**) exhibited average antifungal activity with MIC values of 16, 32, 32, 64 and 64 µg/mL, against *Cladosporium cladosporioides*, *C. cucumerinum*, *Saccharomyces cerevisiae*, *Aspergillus niger* and *Rhizopus oryzae* respectively [63].

Altenusin (**120**) (Figure 6), a biphenyl derivative, was isolated from an endophytic fungus, *Alternaria alternata* Tche-153 of *Terminalia chebula*, collected from Bangkok, Thailand. Employing disk diffusion method and the microdilution checkerboard technique, altenusin (**120**) in amalgamation with each of three azole drugs, ketoconazole, fluconazole or itraconazole at their low sub-inhibitory concentrations displayed potent synergistic activity against *C. albicans* with the fractional inhibitory concentration index range of 0.078 to 0.188 [64]. It is reported that *Schizosaccharomyces pombe* cells treated with altenusin were more rounded in shape than untreated cells which suggest that altenusin could act through the inhibition of cell wall synthesis or assembly in *S. pombe* [65].

Cladosporin (**121**) and isocladosporin (**122**) (Figure 6) were isolated from endophytic fungus *Cladosporium cladosporioides*. Compound **121** exhibited growth inhibition against *Colletotrichum acutatum*, *C. fragariae*, *C. gloeosporioides* and *Phomopsis viticola* at 30 µM with 92.7%, 90.1%, 95.4%, and 79.9%, respectively. Similarly, compound **122** showed 50.4%, 60.2%, and 83.0% growth inhibition against *C. fragariae*, *C. gloeosporioides*, and *P. viticola*, respectively, at 30 µM [66].

Epicolactone (**123**) and epicoccolide A (**124**) and B (**125**) (Figure 6) polyoxygenated polyketides were obtained from an endophytic fungus, *Epicoccum* sp. CAFTBO, associated with stem bark and leaves of *Theobroma cacao* of Mount Kala, Republic of Cameroon. Compounds (**123**–**125**) showed good inhibitory effects on the mycelial growth of *Pythium ultimum* and *Aphanomyces cochlioides* and *Rhizoctonia solani* (MIC in the range of 20–80 µg per paper disc) [67].

5-methylmellein (**100**) (Figure 5) was isolated from endophytic fungus *Biscogniauxia mediterranea* Ohu 19B obtained from *Opuntia humifusa* (Cactaceae) of United States. Antifungal activity of compound **100** was evaluated using an in vitro microdilution broth assay against seven plant pathogens i.e., *Colletotrichum acutatum*, *C. fragariae*, *C. gloeosporioides*, *Fusarium oxysporum*, *B. cinerea*, *Phomopsis obscurans*, and *P. viticola*. *Phomopsis obscurans* was found to be being most susceptible (63.5% growth inhibition) at 150 µM at 120 h. The best growth inhibition (20.1%) to *F. oxysporum* was at 300 µM at 48 h. Lower doses (75 and 150 µM) of this compound caused stimulation of *B. cinera* and *C. fragariae*, while all doses caused stimulation of *C. acutatum* and *C. gloeosporioides* [68].

5-(undeca-3′,5′,7′-trien-1′-yl)furan-2-ol (**126**) and 5-(undeca-3′,5′,7′-trien-1′-yl)furan-2-carbonate (**127**) (Figure 6), two new alkylated furan derivatives, were recovered from the endophytic fungus *Emericella* sp. XL029 associated with the leaves of *Panax notoginseng* collected from Shijiazhuang, Hebei Province, China. Compound **126** displayed good antifungal activity against *Rhizoctorzia solani*, *Verticillium dahliae*, *Helminthosporium maydis*, *Fusarium oxysporum*, *Fusarium tricinctum*, *Botryosphaeria dothidea*, and *Alternaria fragriae* with MIC values ranging from 25 to 3.1 µg/mL, while compound **127** was found active against *V. dahliae*, *H. maydis*, *F.tricinctum*, *B. dothidea*, and *A. fragriae* with MIC values ranging from 50 to 12.5 µg/mL [69].

### 2.3. Compounds Produced by Hyphomycetes

5-hydroxy 2(3H)-benzofuranone (**128**), dehydrocostus lactone (**129**) and harpagoside (**130**) (Figure 6) were isolated from *Fusarium fujikuroi*, *Penicilium chrysogenum* and *Penicillium expensum* endophytes of *Eleusine coracana* grown under semi-hydroponic conditions Arkell Field Station, Arkell, ON, Canada. Compounds **128**–**130** exhibited antifungal activity against *F. graminearum* with the MIC of 31.25, 250.00 and 31.25 µg/mL, respectively. An in vitro interaction between each compound and *Fusarium* was investigated using light microscopy and vitality staining where the results proposed a mixed fungicidal/fungistatic mode of action [70].

Endophytic fungus *Trichoderma koningiopsis* YIM PH30002 harbored in *Panax notoginseng* collected from Wenshan, Yunnan Province, China was the source of koninginin O (**131**), koninginin Q (**132**) and 7-*O*-methylkoninginin D (**133**) (Figure 6). The antifungal activities of these compounds were tested against phytopathogenic fungi, *Fusarium oxysporum*, *F. solani*, *F. flocciferum*, *Plectosphaerella cucumerina* and *Alternaria panax* which are causes of pathogens of root rot diseases of *P. notoginseng*. Koninginin O (**131)** and koninginin Q (**132**) exhibited poor activity against *F. oxysporum* and *P. cucumerina* (MIC of 128 µg/mL). 7-*O*-methylkoninginin D (**133**) also showed poor activity against *P. cucumerina* (MIC 128 µg/mL). Nystatin positive control showed antifungal activity with MICs at 32 µg/mL [71]. Koningiopisin C (**134**) (Figure 6) was also isolated from the same fungus. Koningiopisin C showed antimicrobial activities against *F. oxysporum*, *A. panax*, *F. solani* and *P. cucumerina* with MICs at 32, 64, 32, and 16 µg/mL, respectively [72].

Dichlorodiaportinolide (**135**) and dichlorodiaportin (**136**) (Figure 6) were isolated from endophytic fungus *Trichoderma* sp. 09 obtained from the root of *Myoporum bontioides* A. Dichlorodiaportinolide (**135**) and dichlorodiaportin (**136**) showed weak to high antifungal activities with MIC values ranging from 6.25 to 150 µg/mL against *Colletotrichum musae* and *Rhizoctonia solani* and were inactive to *Penicillium italic* and *Fusarium graminearum* (MIC values > 200 µg/mL) [73].

Trichodermin (**137**) (Figure 6) was isolated from endophytic fungus strain, *Trichoderma brevicompactum* 0248 obtained from *Allium sativum.* Trichodermin showed potent inhibitory activity against *Rhizoctonia solani*, with an EC_50_ of 0.25 µg/mL and against *B. cinerea*, with an EC_50_ of 2.02 µg/mL but relatively poorly active against *Colletotrichum lindemuthianum* (EC_50_ = 25.60 µg/mL). Compound **137** exhibited good antifungal activity against the tested phytopathogens compared with the positive control Carbendazim [74].

**Figure 7:**
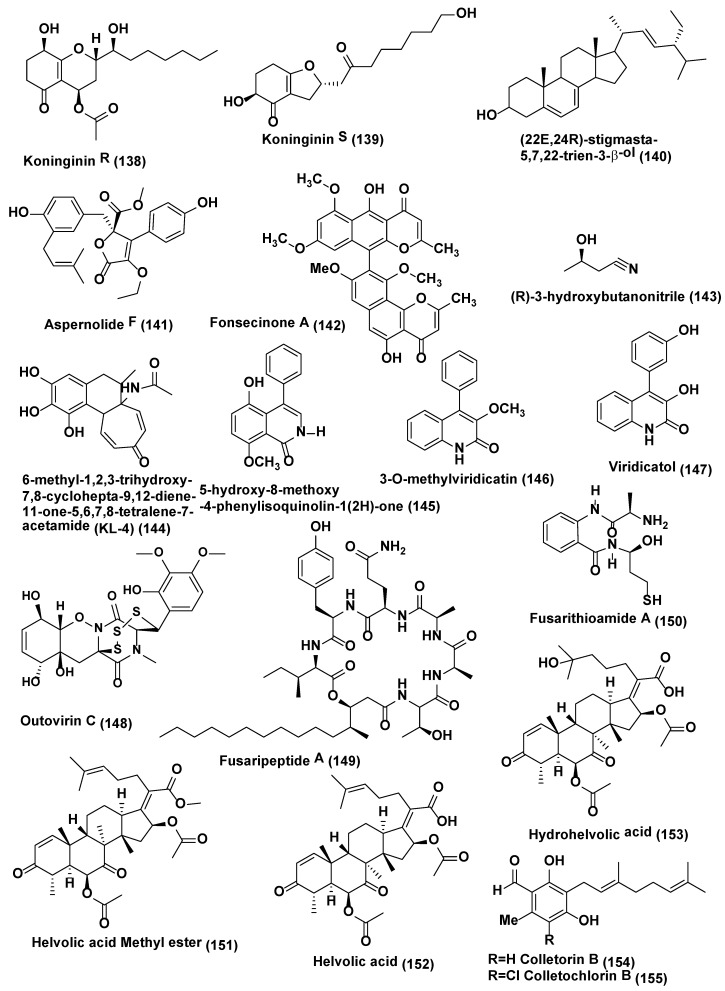
Structures of metabolites isolated from Hyphomycetes (**138**–**155**).

*Trichoderma koningiopsis* YIM PH30002 collected at Wenshan, Yunnan Province of China was the source of two new metabolites koninginins R and S (**138**–**139**) (Figure 7). These isolated compounds showed certain antifungal activities against phytopathogens, *Fusarium flocciferum* and *Fusarium oxysporum*. Compound **138** possess the weak activity against *F. oxysporum* and *F. flocciferum* with the MICs at 128 µg/mL, while compound **139** displayed the poor activity against *F. oxysporum* with the MIC at 128 µg/mL [75].

Stigmasterol derivative (22E,24R)-stigmasta-5,7,22-trien-3-β-ol (**140**) and a new butyrolactones, aspernolide F (**141**) (Figure 7) were obtained from the endophytic fungus *Aspergillus terreus* associated with the roots of *Carthamus lanatus* collected at Assiut, Egypt. Compounds **140**–**141** exhibited good activity against *C. neoformans* with IC_50_ values of 4.38 and 5.19 µg/mL respectively, compared to amphotericin B (IC_50_ 0.34 µg/mL) [76].

Fonsecinone A (**142**), and (R)-3-hydroxybutanonitrile (**143**) (Figure 7), were obtained from *Aspergillus* sp. KJ-9 an endophytic fungus associated with *Melia azedarach* which was collected at Yangling, Shaanxi Province, China, and identified by spectroscopic methods. Compounds **142** and **143** were active against *Gibberella saubinetti*, *Magnaporthe grisea*, *B. cinerea*, *C. gloeosporioides* and *A. solani* (MIC range of 6.25–50 µM) [77].

6-methyl-1,2,3-trihydroxy-7,8-cyclohepta-9,12-diene-11-one-5,6,7,8-tetralene-7-acetamide (KL-4) (**144**) (Figure 7) was isolated from *Aspergillus* sp. obtained from the seeds of *Gloriosa superba* which were collected from Tirupati, India. KL-4 (**144**) exhibited good antifungal activity against *Saccharomyces cerevisiae*, *C. albicans* and *Cryptococcus gastricus* with MIC 25, 12.5, and 50 µg/mL respectively [78].

Endophytic fungus *Penicillium* sp. R22 associated with *Nerium indicum* collected from Qinling Mountain, Shaanxi Province, China was the source of 5-hydroxy-8-methoxy -4-phenylisoquinolin-1(2H)-one (**145**) a new isoquinolone alkaloid along with 3-*O*-methylviridicatin (**146**) and viridicatol (**147**) (Figure 7) two known quinolinone alkaloids. Compound **145** exhibited good antifungal activity against *Alternaria brassicae*, *A. alternata* and *Valsa mali* with MIC value of 31.2 µg/mL, compound **146** against *A. brassicae*, *B. cinerea and Valsa male* with MIC value of 31.2 µg/mL, compound **147** against *A. brassicae*, *A. alternata* and *B. cinerea* with MIC value of 31.2 µg/mL [79].

Trisulfide gliovirin-like compound Outovirin C (**148**) (Figure 7), an epithiodiketopiperazine natural product, was identified from *Penicillium raciborskii*, an endophytic fungus associated with *Rhododendron tomentosum* were collected at the test site of University of Oulu, Finland. Outovirin C (**148**) showed antifungal activity when assayed by micro-spectrophotometry using a dose response growth inhibition assay. Outovirin C inhibited the growth of *Fusarium oxysporum*, *B. cinerea*, and *Verticillium dahlia* at a low concentration of 0.38 mM (207 µg/mL) but a more significant growth inhibition was observed at the higher concentration of 0.76 mM (413 µg/mL). Compound **148** was most active against *B. cinerea* (57% inhibition) and slightly less effective against *V. dahliae* (45% inhibition) [80].

Fusaripeptide A (**149**) (Figure 7), a new cyclodepsipeptide, was isolated from the culture of the endophytic fungus *Fusarium* sp. associated with roots of *Mentha longifolia* growing in Saudi Arabia. Its structure was elucidated based on 1D and 2D NMR and HRESI and GC-MS experiments. The absolute configuration of the amino acid residues of **149** was assigned by chiral GC-MS and Marfey’s analysis after acid hydrolysis. Compound **149** exhibited potent antifungal activity toward *C. albicans*, *C. glabrata*, *C. krusei*, and *A. fumigates* with IC_50_ values of 0.11, 0.24, 0.19, and 0.14 µM, respectively. Under similar condition control amphotericin B exhibited antifungal activity toward *C. albicans*, *C. glabrata*, *C. krusei*, and *A. fumigates* with IC_50_ values of 0.3, 0.6, 0.5, 0.7 µM, respectively [81].

Fusarithioamide A, a new benzamide derivative (**150**) (Figure 7) was isolated from *Fusarium chlamydosporium* associated with the leaves of *Anvillea garcinii* collected from Al-Azhar University, Saudi Arabia. Compound **150** exhibited good antifungal activity against *C. albicans* with inhibition zone diameters (IZD 16.2 mm and MIC 2.6 µg/mL which is comparable to the positive control substance clotrimazole (IZD 18.5 mm and MIC 3.7 µg/mL) [82].

A new helvolic acid derivative named helvolic acid methyl ester (**151**), together with two known helvolic acid compounds, helvolic acid (**152**) and hydrohelvolic acid (**153**) (Figure 7), were extracted from endophytic fungus *Fusarium* sp. associated with *Ficus carica* leaves collected from Qinling Mountain, Shaanxi Province, China. Compounds **151**–**153** exhibited good antifungal activity against *B. cinerea*, *C. gloeosporioides*, *F. oxysporum* f. sp. *niveum*, *Fusarium graminearum* and *Phytophthora capsici* with MIC value in the range of 12.5–25 µg/mL while Carbendazim the standard showed MIC value in the range of 32.2–62.5 µg/mL against the same fungi [83].

**Figure 8:**
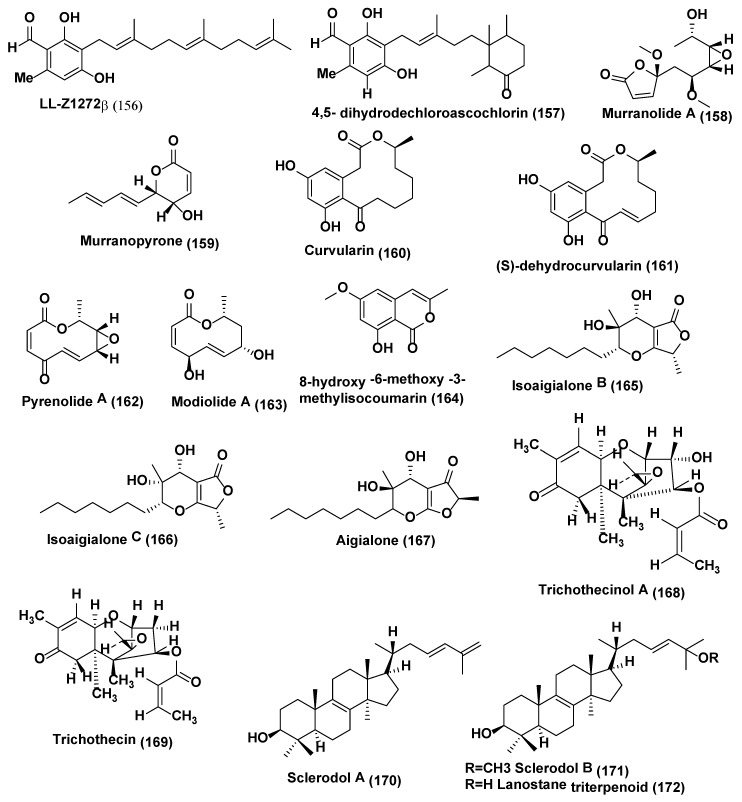
Structures of metabolites isolated from Hyphomycetes (**156**–**169**) and Basidiomycetes (**170**–**172**).

Colletorin B (**154**), colletochlorin B (**155**) (Figure 7), LL-Z1272β (llicicolin B) (**156**), and 4,5-dihydrodechloroascochlorin (**157**) (Figure 8) were extracted from endophytic fungus *Fusarium* sp. Colletorin B (**154**) and colletochlorin B (**155**) showed moderate antifungal activity towards *Ustilago violacea* and *F. oxysporum*. Compound **156** showed moderate antifungal activity towards *U. violacea* and *F. oxysporum*. Furthermore, 4,5-dihydrodechloroascochlorin (**157**) showed a very strong antifungal activity towards *Eurotium repens* [84].

Murranolide A (**158**), murranopyrone (**159**), curvularin (**160**), (S)-dehydrocurvularin (**161**), pyrenolide A (**162**), modiolide A (**163**), and 8-hydroxy-6-methoxy-3-methylisocoumarin (**164**) (Figure 8) were extracted from the endophytic fungus *Curvularia* sp., strain M12, associated with the leaf of *Murraya koenigii* were collected from Rajshahi University, Bangladesh. Pyrenolide A (**162**) was observed to impair the mobility of *Phytophthora capsici* zoospores in a short time (30 min) at a low concentration (100% at 0.5 µg/mL). Murranolide A (**158**), murranopyrone (**159**), curvularin (**160**), (S)-dehydrocurvularin (**161**), modiolide A (**163**), and 8-hydroxy-6-methoxy-3-methylisocoumarin (**164**) exhibited zoospore motility impairment activity at higher concentrations (IC_50_: 50–100 µg/mL) [85].

Two new isoaigialones, B (**165**) and C (**166**) (Figure 8), along with aigialone (**167**) (Figure 8), were obtained from *Phaeoacremonium* sp., an endophytic fungus associated with the leaves of *Senna spectabilis* was collected in the Araraquara Cerrado area, in June 2001, Araraquara, Sao Paulo state, Brazil. These compounds were evaluated against *Cladosporium cladosporioides* and *C. sphaerospermum* using direct bioautography. Compounds **165** and **167** exhibited antifungal activity, with a detection limit of 5 µg, for both fungi, while compound **166** displayed weak activity (detection limit > 5 µg), with a detection limit of 25 µg. Nystatin was used as a positive control, showing a detection limit of 1 µg [86].

Trichothecinol A (**168**) (Figure 8) was obtained from *Trichothecium* sp. an endophytic fungus isolated from *Phyllanthus amarus* collected from Pune India. Compound **101** showed activity against *Cryptococcus albidus* (NCIM 3372) up to 20 µg/mL [87]. Trichothecin **(169)** (Figure 8), a sesquiterpene, was isolated from endophytic fungus *Trichothecium* sp. residing inside the leaves of *Phyllanthus* sp. collected from Pune India. Compound **169** exhibited anti-fungal activity against *Saccharomyces cerevisiae*, *Cryptococcus albidus* var *diffluens* NCIM 3371, *Cryptococcus albidus* var *diffluens* NCIM 3372, *Fusarium oxysporum*, *Penicillium expansum*, *Trichoderma viride*, *Paecilomyces varioti* and *Aspergillus niger* with MIC of 6, 20, 12, 10, 30, 40, 20 and 12 µg/mL, respectively [88].

### 2.4. Compounds Produced by Basidiomycetes

Two lanostane triterpenoids, sclerodols A (**170**) and B (**171**), and a known related lanostane triterpenoid (**172**) (Figure 8) were isolated from *Scleroderma* UFSM Sc1 (Persoon) Fries an endophyte associated with *Eucalyptus grandis*. Both compounds were evaluated for their anti-candidal potential against *Candida albicans*, *C. tropicalis*, *C. crusei*, *C*. *parapsiosis* for activities. Compound **171** showed good anticandidal activity against *C. albicans*, *C. tropicalis*, *C. crusei*, *C. parapsiosis* with the MIC of 25.0, 25.0, 6.25 and 12.5 and MFC of 25.0, 25.0, 12.5 and 25.0 µg/mL respectively. Compounds **170** and **172** were less active against tested strain than compound **171** with the MIC in the range of 12.5–100 and MLC (minimal lethal concentratin) of >100.0 µg/mL. Control nystatin exhibited showed anti-candidal activities against tested strains with the MIC in the range of 0.77–1.52 µg/mL and MLC in the range of 3.12–6.25 µg/mL [89].

## 3. Antifungal Potential of Volatile Organic Compounds (VOCs) from Endophytic Fungi

Volatile organic compounds (VOCs) are generally carbon compounds which exist in the gaseous phase at normal/ambient temperatures and pressures. Over 250 different VOCs produced by fungi comprising different chemical classes such as aldehydes, ketones, alcohols, phenols, thioesters, and so forth, have been identified in the context of the deterioration of fruits, vegetables, indoor environments (sick building syndrome); as chemotaxonomic markers; and in the morphogenesis and development of fungi.

However, bioprospecting fungal endophytes for the production of volatile antimicrobials came into the limelight with the discovery of *Muscodor albus* from the plant *Cinnamomum zeylanicum*, from Honduras. *M. albus* was found to produce an admixture of VOCs which could effectively kill a variety of pathogenic bacteria and fungi associated with plants and animals. This research garnered much attention and drove people to explore the volatile antibiotic properties of endophytic fungi for varied applications [90,91].

The genus *Muscodor* comprises of an endophytic fungi which is predominantly sterile, does not possess true reproductive structures like other fungi, and emanates a characteristic smell which is largely attribute to its VOC composition [92]. Since the report of *M. albus* in the late 1990s, to date, 20 species have been added to this genus, which have largely been identified based on their volatile signatures, molecular phylogeny, and morphological characteristics (Table 2). The characteristic VOC profile, therefore, is helpful in delineating the species, as well as playing a significant role in its anti-fungal and anti-bacterial properties. In this section, we only be highlight the anti-fungal potential of VOCs produced by these endophytic fungi.

The majority of the VOCs produced by the endophytic fungi comprises of a mixture of volatile components which generally has either a synergistic effect or an additive effect that enhances their bioactivity against pathogenic microbes. However, in a couple of studies, the major components of the volatile mixture were independently evaluated to understand their true antimicrobial/anti-fungal potential. These are generally synthetically generated and converted into a volatile form and subsequently evaluated for their bioactivity against the test microorganisms. For instance, *Sclerotina sclerotiorum* was completely inhibited by 2-methyl-1-butanol and 3-methyl-1-butanol with an EC_50_ value of 0.8 µL/mL. 2-methyl-1-butanol also inhibited *Penicillium digitatum* with an EC_50_ value of 0.48 µL/mL and *B. cinerea* with a value of 1.38 µL/mL. However, the volatile admixture of the *M. albus* VOC exhibited an IC_50_ range between 0.08 and 1.13 µL/mL, which clearly confirms the hypothesis of the synergistic/additive effects of the volatile components [93].

Recently, ethyl acetate has been reported to be the main VOC of yeasts *Wickerhamomyces anomalus*, *Metschnikowia pulcherrima*, and *Saccharomyces cerevisiae*, which inhibit the decay causing mold, as well as *B. cineria*. All three yeasts exhibit excellent biological control properties and were used for checking the mold and pathogenic attack in sweet cherries and strawberries. *W. anomalus* induced the highest killing activity amongst the three which was attributed to the higher production of Ethyl acetate. The role of the ethyl acetate was re-affirmed by using synthetic ethyl acetate from strawberry fruits to affirm the anti-fungal action [94].

Similarly, *Phaeosphaeria nodorum*, which existed as an endophyte in plum leaves (*Prunus domestica*) was found to inhibit the pathogen *Monilinia fruticola*. The major component of the VOC produced by *Phaeosphoran odorum* comprised of 3-methyl-1-butanol, acetic acid, 2-propyn-1-ol, and 2-propenenitril [95]. Similarly, six VOCs from the endophytic fungus *Hypoxylon anthochroum* (that is, phenylethyl alcohol), 2-methyl-butanol and 3-methyl-1-butanol, eucalyptol, ocimene, and terpenoline were tested against *Fusarium oxysporum*. The results indicated that these compounds exhibited concentration-dependent anti-fungal activity individually but have better action and control synergistically. Thus, the mixture of six VOCs may be used for the control of *Fusarium oxysporum* in tomatoes [96].

The genus *Muscodor* is one of the best studied endophytic fungus which produces a synergistic mixture of VOCs having lethal effects against a wide variety of plant and human pathogenic fungi, nematodes, and bacteria as well as certain insects [97,98,99,100]. The volatility of the *Muscodor* species has been used to replace methyl bromide (MeBr)—a traditional soil fumigant—which has been globally banned as it causes the depletion of ozone layer. Different species of *Muscodor*, their major VOCs, and their anti-fungal spectrum are given in Table 2. Geographically, each *Muscodor* species has a characteristic signature volatility. For instance, the Indian *Muscodor* species invariably has 4-Octadecylmorpholine as a marker compound while 2-methyl propanoic acid is generally found in Muscodor isolated from North and South America.

The majority of the VOCs from the fungal endophytic fungi are used as biological control agents to prevent the fungal deterioration of crops, fruits, and vegetable, under both pre- and post-harvest conditions. However, the exploitation of these fungally volatile organic compounds (FVOCs) from endophytic fungi are not being actively applied to humans for the prevention of fungal infections.

There exists a huge scope in evaluating these FVOCs from endophytic fungi since they could be helpful in curing superficial skin infections, the sanitization of public toilets, and in night soil. They can also find applications in personal care products such as for the aroma/fragrance in deodorants and sprays. They could presumably be helpful in the development of sprays for inhalation to treat fungal diseases like Aspergillosis in lungs.

## 4. Methods Used for Activation of Silent Biosynthetic Genes

Several research studies confirm that most of the biosynthetic gene clusters are observed to be silent or expressed at a low (minimal) level upon employing conventional culturing conditions for growth/propagation of microorganisms [113]. To activate such silent biosynthetic genes, numerous strategies have been employed, such as the one strain many compounds (OSMAC) approach (activation mediated through modification in composition of medium, aeration, temperature or shape of culturing flask), co-culturing method (facilitating activation through interspecies crosstalk) and genomics based approaches (expression of orphan biosynthesis genes in a heterologous host). In recent times, the use of chemicals as modifiers to alter the epigenetic makeup/constitution of a microorganism to improve its biosynthetic potential has become a beneficial tool. The method uses a chemical that acts as DNA methyltransferase inhibitors (DNMTi) or histone deacetylase inhibitors (HDACi), thereby stimulating the transcription previously silent gene clusters and fostering the production of a spectrum of natural products. A comprehensive description of some of these methods are given below/highlighted in the subsequent section.

### 4.1. Epigenetic Modification

Endophytes have proven to be the prolific source of bioactive metabolites and offer a substitute and untapped reserve for the discovery of novel metabolites. Studies have led to findings that tell biosynthetic gene clusters of microorganisms are mostly silent or expressed at very low levels under standard culture conditions and are least expressed, but under stress condition may it be biological, chemical or physical their expression takes place. Epigenetic modulators lead to the expression of these silent or cryptic genes. Epigenetic gene regulation is mediated by covalent histone modification, DNA methylation chromatin modeling basically induced by DNA methyl transferase inhibitors such as 5-aza-2-deoxycytidine, 5-azacytidine, hydralazine, procaine and histone deacetylase [114]. Chromatic modification in fungi to enhance gene transcription has led to secondary metabolite production of anthraquinones, cladochromes, lunalides, mycotoxins, and nygerones [115]. Structural genes that control transcriptional factor regulates the synthesis of secondary metabolites in fungi, these genes mediate factors occupied in environmental signals like pH, nitrogen and carbon sources, temperature, light, etc. [116]. In lab condition, these gene clusters are mostly silent. Under which natural conditions these clusters become activated is still unexplained. As per genetic sequencing studies carried so far, it is estimated that the clusters of genes responsible for secondary metabolites have not yet been deciphered completely [117].

**Figure 9:**
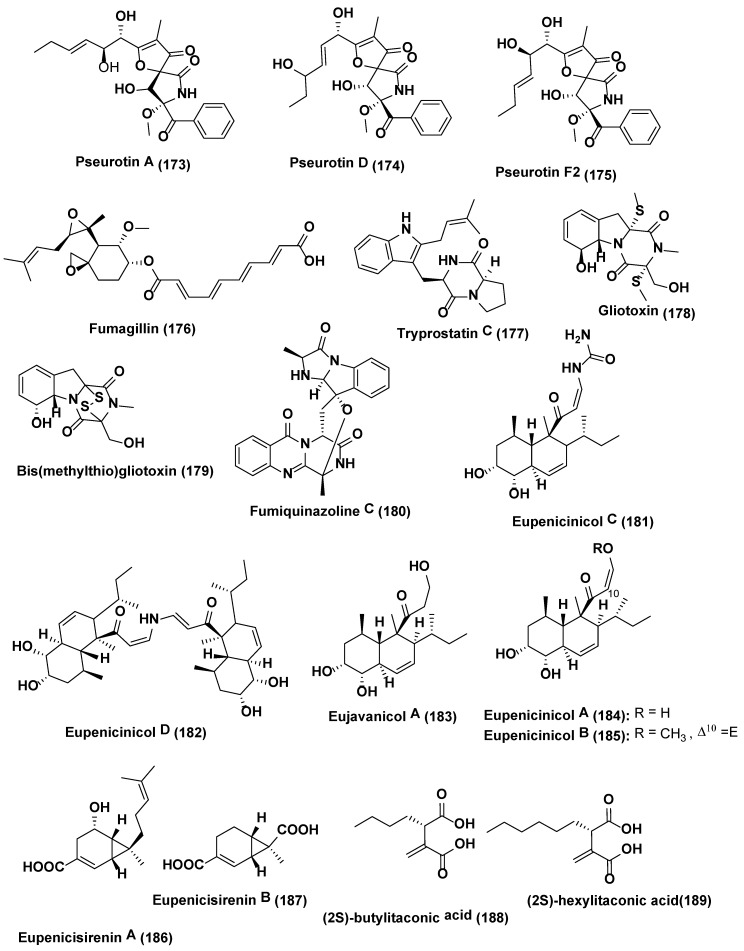
Structures of metabolites isolated from Epigenetic modification in endophytic fungi (**173**–**189**).

From endophytic fungi *Aspergillus fumigatus* (GA-L7) obtained from *Grewia asiatica* led to identification of seven metabolites namely pseurotin A (**173**), pseurotin D (**174**), pseurotin F2 (**175**), fumagillin (**176**), tryprostatin C (**177**), gliotoxin (**178**) and *bis*(methylthio)gliotoxin (**179**) (Figure 9). On addition of the valproic acid, increase in the production of fumiquinazoline C (**180**) up to 10 times was noticed along with a shift in the pattern of metabolite production. It was also observed that all the genes, i.e., Afua_6g 12040, Afua_6g 12050, Afua_6g 12060, Afua_6g 12070 and Afua_6g 12080, tangled in the biosynthesis of fumiquinazoline C (**180**), were upregulated significantly by 7.5, 8.8, 3.4, 5.6 and 2.1 folds, respectively [118].

**Figure 10:**
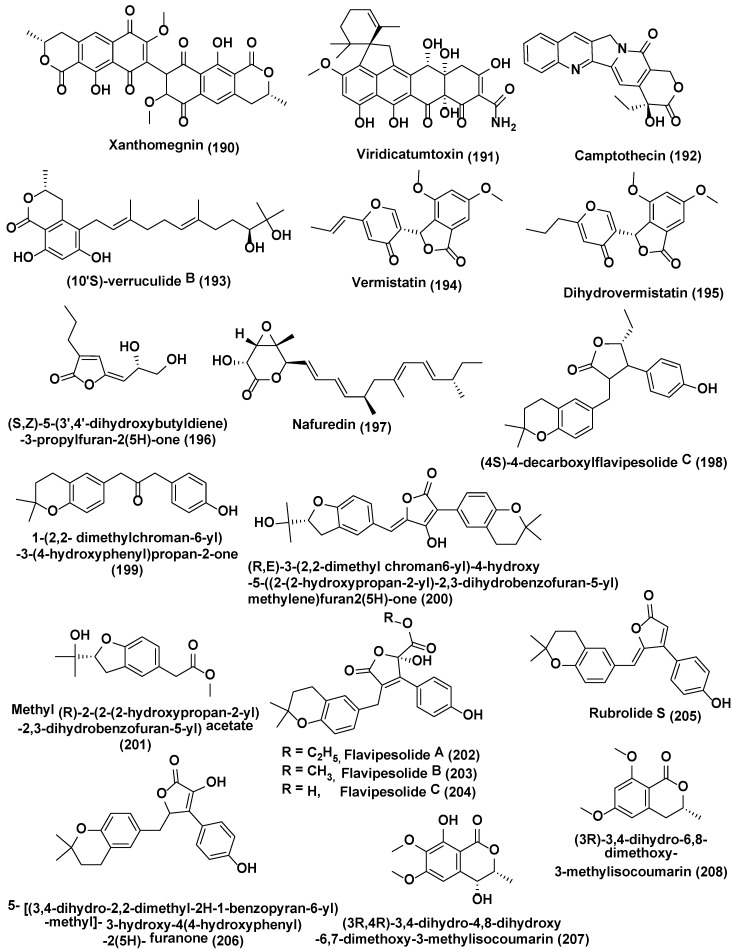
Structures of metabolites obtained from Epigenetic modification in endophytic fungi (**190**–**208**).

The NAD+-dependent HDAC inhibitor, nicotinamide, enhanced the yield of eupenicinicol C (**181**), and D (**182**) (Figure 9), decalin containing metabolites together with eujavanicol A (**183**), and eupenicinicol A (**184**) (Figure 9), biosynthetically related compounds by endophytic *Eupenicillium* sp. LG41, identified from the *Xanthium sibiricum*, a Chinese medicinal plant [119]. Under a similar condition without HDAC inhibitor, nicotinamide *Eupenicillium* sp. LG41 produce different decalin- containing compounds: eupenicinicols A (**184**), and B (**185**); two new sirenin derivatives, eupenicisirenins A (**186**) and B (**187**); and other four known compounds, (2S)-butylitaconic acid (**188**), (2S)-hexylitaconic acid (**189**) (Figure 9), xanthomegnin (**190**), and viridicatumtoxin (**191**) (Figure 10) [120].

Vasanthakumari et al. [121] reported the attenuation of camptothecin (**192**) (Figure 10) yield in endophytic fungi obtained from camptothecin fabricating plants, *Nothapodytes nimmoniana* and *Miquelia dentata* incorporated with 5-azacytidine, a DNA methyltransferase inhibitor. It was indicated that the mode behind decrease of camptothecin yield in endophytic fungi could in principle be inverted by stimulating some signals from the tissue of the plant, probably the methylation or silencing of the genes liable for camptothecin production.

Metabolites such as (10′S)-verruculide B (**193**), vermistatin (**194**), and dihydrovermistatin (**195**) (Figure 10) were identified owing to the supplementation of HDAC inhibitor, suberoylanilide hydroxamic acid (SAHA) to endophytic fungi culture *Phoma* sp. nov. LG0217 isolated from *Parkinsonia microphylla*. However, in absence of SAHA, a novel metabolite (S,Z)-5-(3′,4′-dihydroxybutyldiene)-3-propylfuran-2(5H)-one (**196**), along with nafuredin (**197**) (Figure 10), was produced [122].

**Figure 11:**
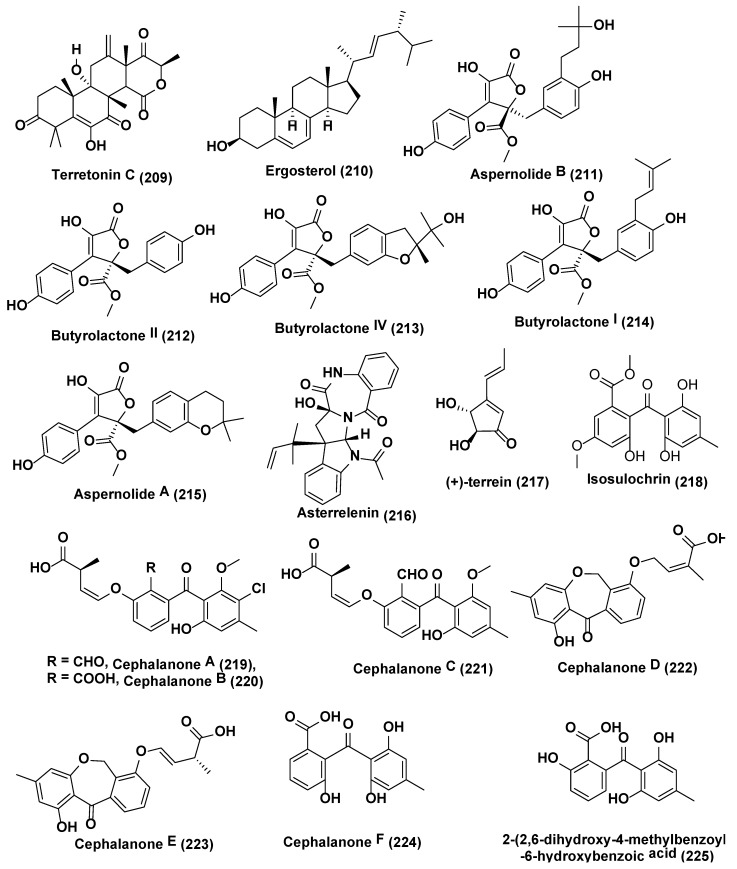
Structures of metabolites obtained from Epigenetic modification in endophytic fungi (**209**–**225**).

Four new meroterpenoids identified as (4S)-4-decarboxylflavipesolide C (**198**), 1-(2,2-dimethylchroman-6-yl)-3-(4-hydroxyphenyl)propan-2-one **(199)**, (R,E)-3-(2,2-dimethyl chroman6-yl)-4-hydroxy-5-((2-(2-hydroxypropan-2-yl)-2,3-dihydrobenzofuran-5-yl)methylene)furan2(5H)-one (**200**), methyl (R)-2-(2-(2-hydroxypropan-2-yl)-2,3-dihydrobenzofuran-5-yl) acetate (**201**), along with nine known compounds flavipesolides A−C (**202–204**), rubrolide S (**205**), 5-[(3,4-dihydro-2,2-dimethyl-2H-1-benzopyran-6-yl)-methyl]-3-hydroxy-4(4-hydroxyphenyl)-2(5H)-furanone (**206**), (3R,4R)-3,4-dihydro-4,8-dihydroxy-6,7-dimethoxy-3-methylisocoumarin (**207**), (3R)-3,4-dihydro-6,8-dimethoxy-3-methylisocoumarin (**208**) (Figure 10), terretonin C (**209**), and ergosterol (**210**) (Figure 11) were obtained using chemically modified epigenetic culture of *Aspergillus terreus* OUCMDZ-2739 with 10 µM trichostatin A (TSA). Under the similar parameters without TSA, *A. terreus* OUCMDZ-2739 yielded many compounds, i.e., aspernolide B (**211**), butyrolactone II (**212**), butyrolactone IV (**213**), butyrolactone I (**214**), aspernolide A (**215**), asterrelenin (**216**) and (+)-terrein (**217**) (Figure 11), supporting that fungal metabolite enrichment and chemodiversity using epigenetic modifiers can be done to obtain new products [123]. Induction of isosulochrin **(218)** (Figure 11) was also witnessed when *Chaetomium* sp. was supplemented with 5-azacytidine or SAHA on solid rice medium [124].

Asai et al. [125] reported six new benzophenones, cephalanones A–F (**219**–**224**), and 2-(2,6-dihydroxy-4-methylbenzoyl)-6-hydroxybenzoic acid (**225**) (Figure 11) from culture of *Graphiopsis chlorocephala*, from *Paeonia lactiflora* in presence of HDAC inhibitors nicotinamide (10 µM) which resulted in significant increase in secondary metabolite production.

To express silent biosynthetic pathways, molecules such as HDAC and DNMT are used to enhance the fungal metabolites production. Different studies suggest an increase in chemical diversity of metabolites by induction with these epigenetic modifiers. For growth and acclimatization with the environment fungus are known to produce diverse secondary metabolites. Cross talk between microbes and plant lead to the expression of these pathways which stays silent in in vitro conditions. Metabolic profiles shift led by SMs induced modifier is due to expression of cryptic genes [126].

### 4.2. The Co-Culture Strategy

Interspecific interaction among different species leads to evolution and biodiversity, organism combines their genetic information for better adaptability. The cohabitation of different microorganisms that share similar niches competes with growth, morphology, adaptation, and development patterns [127,128]. The increased productions of metabolites in co-culture which are not produced in axenic culture are the result of competition or antagonism faced by the microorganism that leads to activation of cryptic genes. [129]. Co-cultivation is a way to provide natural habitat to fungi so that gene clusters become activated. In *Aspergillus nidulans*, the cryptic gene has been successfully activated leading to isolation of novel compounds [130].

**Figure 12:**
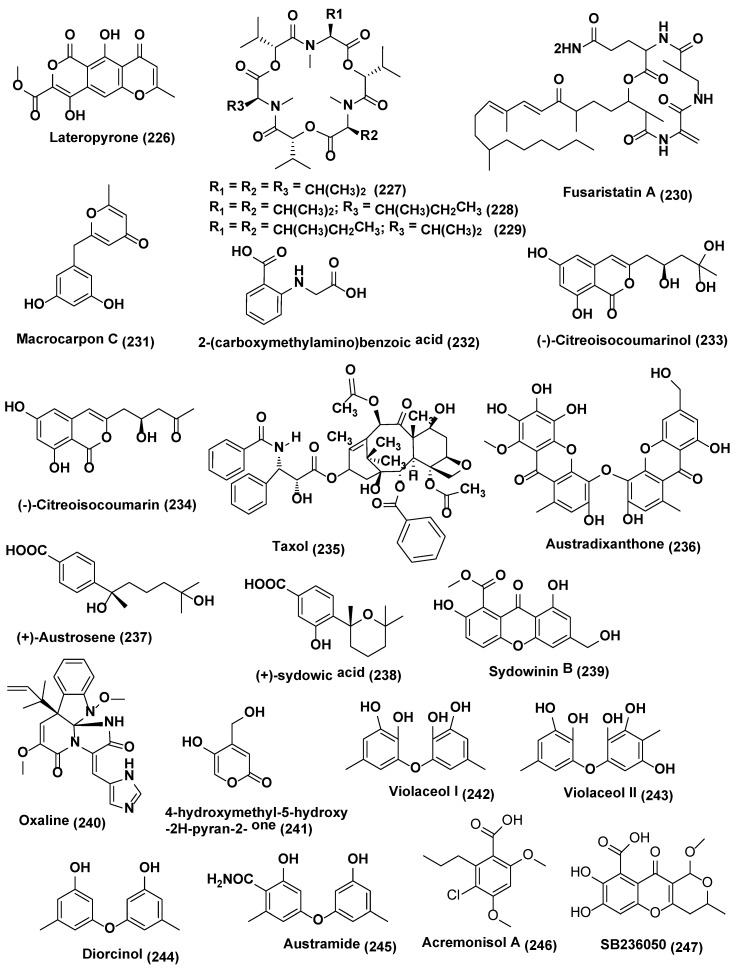
Structures of metabolites obtained from co-culture in endophytic fungi (**226**–**247**).

In a study carried by Ola et al. [131], accumulation of secondary metabolites, i.e., lateropyrone (**226**), cyclic depsipeptides of the enniatin type (**227**–**229**), and the lipopeptide fusaristatin A (**230**) (Figure 12), was found to be enhanced by 78 folds by co-culturing *B. subtilis* 168 trpC2 with *Fusarium tricinctum* isolated from the *Aristolochia paucinervis.* This led to the identification of three new compounds macrocarpon C (**231**), 2-(carboxymethylamino)benzoic acid (**232**) and (−)-citreoisocoumarinol) (**233**), and a known compound, (−)-citreoisocoumarin (**234**), which was absent in axenic culture of bacterial or fungal control. On coculturing *Alternaria* sp. and *Phomopsis* sp. there was the enhancement of taxane (**235**) production by eight fold [132].

**Figure 13:**
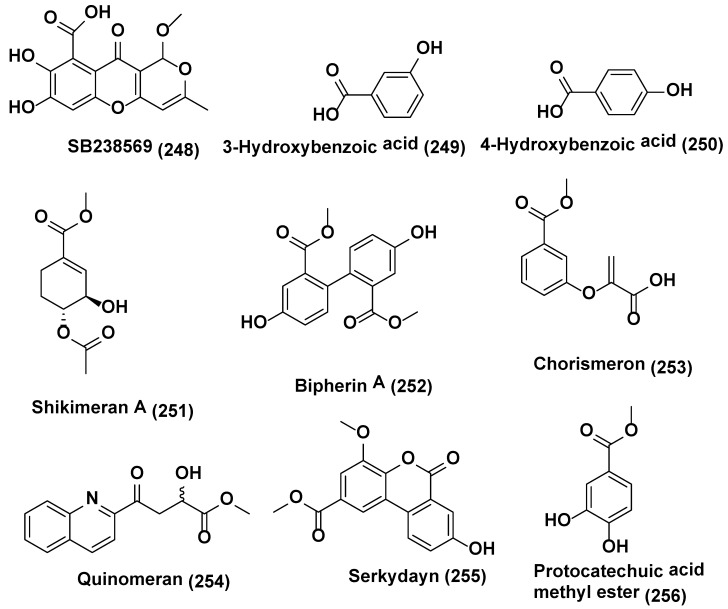
Structures of metabolites obtained from co-culture in endophytic fungi (**248**–**256**).

*Aspergillus austroafricanus* endophyte residing inside the leaves of *Eichhornia crassipes* was the source of two new metabolites, namely, xanthone dimer austradixanthone (**236**) and sesquiterpene (+)-austrosene (**237**), and five known compounds, (+)-sydowic acid (**238**), sydowinin B (**239**), oxaline (**240**), 4-hydroxymethyl-5-hydroxy-2H-pyran-2- one (**241**) (Figure 12), ergosterol (**210**) (Figure 13). However, the same endophyte grown in mixed cultures with *Bacillus subtilis* or *Streptomyces lividans* led to the identification of many diphenyl ethers i.e., violaceol I (**242**), violaceol II (**243**), and diorcinol (**244**) (Figure 12) along with new austramide (**245**) (Figure 12), increased up to 29 times [133].

*Chaetomium* sp. was isolated from *Sapium ellipticum* the Cameroonian medicinal plant. When *Chaetomium* sp. was cultured axenically on solid rice medium, average yields per culture flask were 2.8, 13.9, 132.7 and 14.6 mg of acremonisol A (**246**), SB236050 (**247**) (Figure 12), and SB238569 (**248**), respectively, and 1:1 mixture of 3- and 4-hydroxybenzoic acid methyl esters (**249–250**), respectively, (Figure 13) was observed. When Co-cultivation of *Chaetomium* sp. was undertaken with viable or autoclaved cultures of *Bacillus subtilis* there was a strong accumulation of the 1:1 mixture of (**249**), and (**250**), was observed, accounting for an 8.3 and 7.4-fold increase, respectively, compared to axenic fungal controls in both cases. SB236050 (**247**) and SB238569 (**248**), two major polyketides of *Chaetomium* sp., were not detected in co-cultures. Five new compounds, Shikimeran A (**251**), Bipherin A (**252**), Chorismeron (**253**), Quinomeran (**254**), and Serkydayn (**255**), and two known compounds, isosulochrin **(218)** and protocatechuic acid methyl ester (**256**) (Figure 12), were only detected in co-cultures of *Chaetomium* sp. with viable or autoclaved *B. subtilis* cultures, but were lacking in both fungal or bacterial controls when cultured axenically [124].

These studies indicate that co-culture generates a complex and promising environment to obtain new secondary metabolites as a response to the interaction between endophytic fungi. The above also indicates that the production of new natural products depends on stimuli.

## 5. Conclusions

Endophytic fungi are the ubiquitous source of novel chemical compounds having the potential to display antifungal activities. Interestingly, the active metabolites from endophytic fungi possess excellent antifungal activity not only against human fungal pathogens but also on plant fungal pathogens. In addition, the volatile organic compounds (VOCs) from genus *Muscodor* displayed significant antifungal as well as antibacterial properties and, therefore, they are used to prevent fungal deterioration of crops, fruits and vegetables. However, their application to control human fungal infection has not been explored. Fungal VOCs can be investigated for the development of sprays for inhalation to treat fungal diseases such as Aspergillosis in lungs, curing superficial skin infections and sanitization. Endophytic fungi are being studied to produce natural compounds which are originally produced from their host plants and, thus, emerging as an alternative and sustainable source of valuable natural products. It is important to investigate the interactions between endophytic fungi with the host plant and other endophytes which are very sensitive to the culture conditions and hence, provide an opportunity to tune the in vitro culture conditions to produce the desired range of secondary metabolites. It is possible to produce a compound of interest by varying the culture conditions such as media composition, aeration rate and temperature. In addition, cultivation of endophytic fungi in presence of bacteria or other fungi (co-cultivation) yield novel compounds which otherwise do not appear when fungi or bacteria are cultivated alone. Therefore, considerable research on endophytic fungi is required for the development of suitable co-culture system for the sustained production of the desired secondary metabolite.

## Figures and Tables

**Table 1:** Antifungal compounds reported from endophytic fungi.

Sr. No.	Fungus	Plantsource	Compounds Isolated	Biologicalactivity *	Refs.
	**Comounds Produced by Coelomycetes**				
1	*Pestalotiopsis foedan*	*Bruguiera sexangula* Hainan, China	(3R,4R,6R,7S)-7-hydroxyl-3,7-dimethyl-oxabicyclo[3.3.1]nonan-2-one (**1**), (3R,4R)-3-(7-methylcyclohexenyl)-propanoic acid (**2**)	Compound **1** *B. cinerea* and *P. nicotianae* (MIC 3.1 and 6.3 µg/mL), ketoconazole (MIC 3.1 µg/mL each) Compound **2** *C. albicans* MIC 50 µg/mL) ketoconazole (MIC 6.3 µg/mL)	[15]
2	*Pestalotiopsis* sp. DO14	*Dendrobium officinale*, Yandang Mountain, Zhejiang Province, China.	(4S,6S)-6-[(1S,2R)-1,2-dihydroxybutyl]-4-hydroxy-4-methoxytetrahydro-2H-pyran-2-one (**3**) and (6S,2E)-6-hydroxy-3-methoxy-5-oxodec-2-enoic acid (**4**), LL-P880γ (**5**), LL-P880α (**6**)	Compounds **3**–**6** active against *C*. *albicans*, *C. neoformans*, *T. rubrum*, *and A. fumigates* (MIC ≤ 50 µg/mL) Compounds **3**–**4** active against *C. albicans*, *C. neoformans*, *T. rubrum*, and *A. fumigatus* (MIC, ≤ 25 µg/mL)	[16]
3	*Pestalotiopsis fici*	*Camellia sinensis* Hangzhou, China.	Ficipyrone A (**7**)	Compound **7** active against *G. zeae* (IC_50_ 15.9 µM), ketoconazole (IC_50_ 6.02 µM)	[17]
4	*Pestalotiopsis mangiferae*	*Mangifera indica* Maduravoyal, Tamil Nadu Province, India.	4-(2,4,7-trioxa-bicyclo[4.1.0]heptan-3-yl) phenol (**8**)	Compound **8** active against *C. albicans* (MIC, 0.039 µg/mL), Nystatin (MIC 10.0 µg/mL)	[18]
5	*Phomopsis* sp.	*Senna spectabilis* São Paulo, Brazil	Cytochalasin H (**9**)	Compound **9** active against *C. cladosporioides* and *C. sphaerosphermum* (MIC 10.0 and 25.0 µg, respectively), nystatin (MIC = 1.0 µg)	[19]
6	*Phomopsis* sp.	*Aconitum carmichaeli*, Huize County, Yunnan Province, China.	(14β,22E)-9,14-dihydroxyergosta-4,7,22-triene-3,6-dione (**10**), (5α,6β,15β,22E)-6-ethoxy-5,15-dihydroxyergosta-7,22-dien-3-one (**11**), calvasterols A (**12**), and ganodermaside D (**13**)	Compound **10** active against *C. albicans*, *H. compactum*, and *A. niger*, (MIC, 64, 64, and 128 µg/mL, respectively). Compound **11** active against *C. albicans* and *F. avenaceum* (MIC = 128 µg/mL). Compounds **12** and **13** active against *F. avenaceum*. (MIC, 64 µg/mL), Compound **12** activie against *P. oryzae* and *T. gypseum* (MIC 128 and 256 µg/mL)	[20]
7	*Diaporthe maritima*	*Picea* sp., Acadian forest of Eastern Canada.	Phomopsolide A (**14**), B (**15**), and C (**16**), and a stable alpha-pyrone (**17**)	Compound **14** active against *M. violaceum* and *S. cerevisiae* at 25 µM, Compounds **15**–**17** demonstrated growth inhibition at 250 µM	[21]
8	*Phoma* sp.	*Fucus serratus*,	Phomalacton (**18**), (3R)-5-hydroxymellein (**19**) and emodin (**20**)	Compounds **18**–**20** active against *M. violaceum* with 5, 6 and 5mm zone of inhibition.	[23]
9	*Phoma* sp. WF4	*Eleusine coracana* Arkell Field Station, Arkell, ON, Canada	Viridicatol (**21**), tenuazonic acid (**22**), alternariol (**23**), and alternariol monomethyl ether (**24**)	Compounds **21**–**24** caused dramatic breakage of *F. graminearum* hyphae in vitro	[24]
10	*Rhizopycnis vagum* Nitaf 22	*Nicotiana tabacum*, China Agricultural University. Beijing 100193, China.	Rhizopycnin D (**25**) and TMC-264 (**26**)	Compounds **25**–**26** inhibited the spore germination of *M. oryzae* with IC_50_ values of 9.9 and 12.0 µg/mL, respectively	[25]
11	*Microsphaeropsis* sp. *Seimatosporium* sp.	*Salsola oppositifolia*, Playa del Ingles, Gomera, Spain	Microsphaerol (**27**) Seimatorone (**28**)	Compounds **27** and **28** active against *M. violaceum* with 9 and 5 mm zone of inhibition. In addition, there was some growth with in zone of inhibition	[26]
12	*Colletotrichum gloeosporioides*	*Michelia champaca* São Paulo State University, Araraquara, São Paulo, Brazil.	2-phenylethyl 1H-indol-3-yl-acetate (**29**)	Compound **29** active against *C. cladosporioides* and *C. sphaerospermum* comparable to that of the positive control nystatin	[27]
13	*Colletotrichum* sp.	Gomera (Spain).	Colletonoic acid (**30**)	Compound **30** active against *M. violaceum* with 7 mm zone of inhibition	[28]
14	*Coniothyrium* sp.,	*Salsola oppostifolia* Gomera in the Canary Islands.	1,7-dihydroxy3-methyl-9,10-anthraquinone (**31**), 1,6-dihydroxy-3 -methyl-9,10-anthraquinone (phomarin) (**32**), and 1-hydroxy-3-hydroxymethyl-9,10-anthraquinone (**33**) coniothyrinones A-D (**34**–**37**)	Compounds **31**–**37** active against *M. violaceum* with 7, 10, 8, 7.5, 6, 8 and 7.5 mm zone of inhibition. Compounds **32**–**34** active against *M. violaceum* (10 and 9 mm zone of inhibition) and *B. cinerea* (7.5 and 12.5 mm zone of inhibition) when tested under similar conditions	[29,30]
	**Comounds Produced by Acsomycetes**				
15	*Xylaria* sp. YM 311647	*Azadirachta indica*,Yuanjiang County, Yunnan Province, China,	(1S,4S,5R,7R,10R,11R)-Guaiane-5,10,11,12-tetraol (**38**) (1S,4S,5S,7R,10R,11S) -Guaiane-1,10,11,12-tetraol (**39**) (1S,4S,5R,7R,10R,11S)-Guaiane-5,10,11,12-tetraol (**40**) (1S,4S,5S,7R,10R,11R)-Guaiane-1,10,11,12-tetraol (**41**) (1R,3S,4R,5S,7R,10R,11S) -Guaiane-3,10,11,12-tetraol (**42**) (1R,3R,4R,5S,7R,10R,11R)-Guaiane-3, 10,11,12-tetraol (**43**) (1R,4S,5S,7S,9R,10S,11R)-Guaiane-9,10,11,12-tetraol (**44**) (1R,4S,5S,7R,10R,11S) -Guaiane-10,11,12-triol (**45**) (1R,4S,5S,7R,10R,11R)-Guaiane-10,11,12-triol (**46**), 14a,16-Epoxy-18-norisopimar-7-en-4a-ol (**47**),16-*O*-Sulfo-18-norisopimar-7-en-4a,16-diol (**48**), and 9-Deoxy-hymatoxin A (**49**)	Compounds **38**–**46** active against *C. albicans* and *H. compactum* (MIC in the range of 32 to 256 µg/mL), compounds **47**–**49** active against *C. albicans*, *A. niger*, *P. oryzae*, *F. avenaceum*, and *H. compactum* (MIC in the range of 16 to 256 µg/mL). Compound **49** exhibited the potent inhibitory activity against *C. albicans* and *P. oryzae* with MIC values of 16 µg/mL	[32]
16	*Xylaria* sp. YM 31164	*Azadirachta indica*, Yuanjiang County, Yunnan Province, China	(1S,2S,4S,5S,7R,10R)-Guaiane-2,10,11,12-tetraol (**50**), (1S,2S,4R,5R,7R,10R)-Guaiane-2,4,10,11,12-pentaol (**51**), (1S,4R,5S,7R,10R)-Guaiane-4,5,10,11,12-pentaol (**52**), (1R,4S,5R,7R,10R)-Guaiane-1,5,10,11,12-pentaol (**53**), (1R,4R,5R,7R,10R)-11-Methoxyguaiane-4,10,12-triol (**54**),	Compounds **50**–**54** active against *P. oryzae* and *H. compactum* (MIC in the range of 32–256 µg/mL). Compound **53** active against *P. oryzae* (MIC 32 µg/mL). Compounds **52** and **53** active against *H. compactum* with (MIC, 64 µg/mL), Compound **53** and **54** active against *C. albicans* (MIC 32 µg/mL). Compound **52** active against *C. albicans*, *A. niger*, and *H. compactum* (MIC, 64 µg/mL).	[33]
17	*X. feejeensis*	*Croton lechleri*.	Xyolide (**55**),	Compound **55** active against *P. ultimum* (MIC 425 µM)	[34]
18	*Xylaria* sp. XC-16	*Toona sinensis* Yangling, Shaanxi Province, China	Cytochalasin Z28 (**56**)	Compound **56** active against *G. saubinetti* (MIC of 12.5 µM), Hymexazol (MIC = of 25 µM)	Zhang et al. [35]
19	*Xylaria* sp. strain F0010, *Xylaria* sp. PSU-G12*X. cubensis*, 13 strains of *Xylaria* sp.	*Abies holophylla; Garcinia hombroniana ; Asimina triloba; Pinus strobus; Vaccinium angustifolium*, New Brunswick and Nova Scotia, Canada	Griseofulvin (**57**)	Griseofulvin (**57**) Inhibits *A. mali*, *B. cinerea*, *Colletotrichum gloeosporioides*, *Corticium sasaki*, *F. oxysporum* and *M. grisea* in in vitro (IC_50_ values of 18.0, 5.0, 1.7, 11.0, 30.0, and 1.7 µg/mL, respectively, Compound **57** active against *M. grisea*, *C. sasaki*, *B. cinerea*, *P. recondite* and *B .graminis* f. sp. *hordei* in in vivo, with % of fungal control of 95, 100, 60, 90 and 90, respectively, at 150 µg/mL.	[36,37,38]
20	*Xylaria* sp.		Dechlorogriseofulvin (**58**)	Compound **58** showed weak antifungal activity, with an IC_50_ value, 200 µg/mL against *M. grisea*, *C. sasaki*, *B. cinerea*, *P. recondite* and *B .graminis* f. sp. *hordei* in in vivo,	[36,38]
21	*Chaetomium globosum* CDW7	*Ginkgo biloba* China	Chaetoglobosin A (**59**) and D (**60**)	Compounds **59**–**60** active against *S. sclerotiorum* with IC_50_ values of 0.35 and 0.62 µg/mL, respectively, carbendazim (0.17 µg/mL)	[41]
22	*Chaetomium globosum*	Seeds of *Panax notoginseng* collected at the Wenshan, Yunnan, China	Chaetoglobosin A (**59**), Chaetomugilin A (**61**), Chaetomugilin D (**62**), Chaetoglobosin B (**63**), Chaetoglobosin E (**64**), Chaetoglobosin F (**65**) **and** Penochalasin G (**66**)	Compounds **59** and **61**–**66** active against *P. herbarum* (MIC in the range of 16–128 µg/mL) and, *E. nigrum* (MIC in the range of <1–16 µg/mL).	[42]
23	*Chaetomium cupreum* ZJWCF079	*Macleaya cordata.*	Ergosta-5, 7, 22-trien-3-beta-ol (**67**)	Compound **67** against *S. sclerotiorum* and *B. cinerea* with EC_50_ values of 125 µg/mL and 190 µg/mL respectively,	[43]
24	*Chaetomium globosum* No.04	Barks of *Ginkgo biloba*, Linyi, Shandong Province, China.	Chaetoglobosin A (**59**), D (**60**), E (**64**), C (**68**), G (**69**), R (**70**)	Compounds **59**–**60**, **64**, and **68**–**70** active against *R. stolonifer* and *C. diplodiella* at a concentration of 20 µg/disk	[44]
25	*Botryosphaeria* sp. P483	*Huperzia serrata*, Xichou County, Yunnan Province, China	Botryosphaerin H (**71**) 13,14,15,16-tetranorlabd-7-en-19,6β:12,17-diolide (**72**)	At 100 µg/disk, compound **71** showed zone of inhibition of 9, 7, 7, 8, and 8 mm, against *G. graminis*, *F. solani*, *P. oryzae*, *F. moniliforme*, and *F. oxysporum*; compound **72** showed zone of inhibition of 12, 10, 10, 11, and 13 mm against *G. graminis*, *F. solani*, *P. oryzae*, *F. moniliforme*, and *F. oxysporum;* carbendazim (50 µg/disk) showed the zone of inhibition of 14, 18, 15, 17, 15 mm against *G. graminis F. solani P. oryzae F. moniliforme F. oxysporum*, respectively	[45]
26	*Botryosphaeriadothidea* KJ-1,	*Melia azedarach* Yangling, Shaanxi Province, China.	Pycnophorin (**73**), stemphyperylenol (**74**), chaetoglobosin C (**68**), djalonensone (**75**), alternariol (**76**), β-sitosterol glucoside (**77**), 5-hydroxymethylfurfural (**78**)	Compound **74** active against *A. solani* (MICs of 1.57 µM) Compounds **68**, **73**, and **75**–**78** active against *A. solani* (MICs of 6.25−25 µM)	[46]
27	*Mycosphaerella* sp.	*Eugenia bimarginata* DC. Brazil (savannah).	2-amino-3,4-dihydroxy-2-25-(hydroxymethyl)-14-oxo-6,12-eicosenoic acid (**79**), myriocin (**80**)	Compounds **79** active against several isolates of *C. neoformans* and *C*. *gattii*, with MIC values ranging from 1.3 to 2.50 µg/mL and 0.5 µg/mL, for compound **80**	[47]
				**Compounds 79** active against several isolates of *C*. *neoformans* and *C. gattii*, with MIC values ranging from 0.49 to 7.82 µM and 0.48–1.95 µM for compound **80**. Compounds **79** and **80** cause deformities in cell shape, depressions on the surface, and withered cells.	[48]
28	*Guignardia* sp.,	*Euphorbia sieboldiana* collected from the campus of China Pharmaceutical University, Nanjing, Jiangsu, China	Guignardone N (**81**), guignardic acid (**82**)	At 6.3 µg/mL combined with 0.031 µg/mL of fluconazole, compounds **81** and **82** were found to have prominent inhibition on the growth of *C. albicans* with FIC index values of 0.23 and 0.19, respectively. Combined with fluconazole, both of them (40 µg/mL for (**81**) and 20 µg/mL for (**82**) could also inhibit *C. albicans* biofilms and reverse the tolerance of *C. albicans* biofilms to fluconazole	[49]
29	*Hyalodendriella* sp. Ponipodef 12	“Neva” hybrid of *Populus deltoides* Marsh × *P. nigra* L., Longhua in Hebei Province of China.	hyalodendriol C (**83**), rhizopycnin D (**84**), palmariol B (**85**), TMC-264 (**86**), penicilliumolide B (**87**) and alternariol 9-methyl ether (**88**)	Compound **88** exhibited spore germination of *M. oryzae* with IC_50_ value of 11.6 µg/mL, positive control, carbendazim (IC_50_ 6.9 µg/mL) Compounds **84**–**88** displayed antifungal effects against the spore germination of *M. oryzae*	[50,51,52]
30	*Pezicula* sp.	*Forsythia viridissima*, collected from Zhejiang Province, Southeast China	Mellein (**89**)	Compound **89** active against *B. cinerea*, *P. ultimum*, *F. oxysporium f.* sp. *cucumerinum*, *C. orbiculare*, *V. dahliae*, *P. oryzae*, *P. diospyri*, *S. sclerotiorum* and *F. fulva*, especially *B. cinerea* and *F. fulva* with EC_50_ values below 50 µg/mL	[53]
31	*Nodulisporium* sp. A21	Leaves of *Ginkgo biloba*. Nanjing in Jiangsu Province, China	Sporothriolide, (**90**)	The EC_50_ of compound **90** against *R.solani* was 3.04 µg/mL (11.6 µM), while the EC_50_ of carbendazim was 1.84 µg/mL (9.6 µM).	[54]
32	*Lopherdermium nitens* DAOM 250027	*Pinus strobus* Sussex, NB, Canada	Six phenolic bisabolane-type sesquiterpenoids (**91**–**96**), pyrenophorin (**97**)	Compound **97** significantly reduced the growth of *M. violaceum* and *S. cerevisiae* at 5 µM whereas sesquiterpenoids **91**–**96** active at 50 µM to both species tested	[55]
33	*Exserohilum* sp.	*Acer truncatum* Beijing, China.	Exserolide C (**98**), (12R)-12-hydroxymonocerin (**99**)	Compounds **98** and **99** active against *F. oxysporum*, both showing a MIC value of 20 µg/mL, Amphotericin B (MIC, 0.63 µg/mL)	[56]
34	*Biscogniauxia mediterranea* EPU38CA	*Echinacea purpurea* Missouri, USA,	(−)-5-methylmellein (**100**) and (−)-(3R)-8-hydroxy-6-methoxy-3,5-dimethyl-3, 4-dihydroisocoumarin (**101**)	Compound **100** active against *P. obscurans*, *P. viticola*, and *F.oxysporum*, and caused growth stimulation of *C. fragariae*, *C. acutatum*, *C. gloeosporioides*, and *B. cinerea*. Compound **101** was found to be slightly more active in the microtiter environment than 5-methylmellein	[57]
35	*Hypoxylon submonticulosum*	*Rubus idaeus* collected from Jordan Station, ON, Canada.	Trienylfuranol A (**102**) Complete hydrogenation of (**102**) yielded THF 7 (**103**)	THF 7 (**103**) inhibited the growth of *S. cervisiae* (74 ± 4% inhibition) at a concentration of 250 µg/mL as compared with complete inhibition by nystatin at 10 µg/mL	[58]
36	*Phialophoramustea*	*Crocus sativus*.	Phialomustin C-D (**104**) (**105**)	Compounds **104**–**105** active against *C. albicans* (IC_50,_ 14.3 and 73.6 µM)	[59]
37	unidentified Ascomycete,	*Melilotus dentatus*.	*cis*-4-acetoxyoxymellein (**106**) and 8-deoxy-6-hydroxy-*cis*-acetoxyoxymellein (**107**)	Compounds **106** and **107** displayed activities toward *M. violaceum*, *B. cinerea*, with 8 mm zone of inhibition for both fungi.	[60]
38	*Plectophomella* sp.		(−)-Mycorrhizin A (**108**)	Compound **108** active against *U. violacea* and *E. repens*.	[61]
39	*Physalospora* sp.		Cytochalasin E (**109**) and K (**110**)	Compound **109**–**110** active against *E. repens* and *M. microspora*	[61]
40	*Crataegus monogyna.*		Radicinin (**111**)	Radicinin (**111**) active against *E. repens* and *M. microspora*	[61]
41	*Berkleasmium* sp.,	*Dioscorea zingiberensis*. Hubei Province, China.	Diepoxin ζ (**112**), palmarumycin C11 (**113**), palmarumycin C12 (**114**), cladospirone B (**115**), palmarumycin C6 (**116**), 1,4,7β-trihydroxy-8-(spirodioxy-1′,8′-naphthyl)-7,8-dihydronaphthalene (**117**) and palmarumycin C8 (**118**)	Compounds **112**–**118** inhibited spore germination of *M. oryzae* (IC_50_ values in the range 9.1−124.5 µg/mL). Compound **118** showed the best inhibitory activity (IC_50,_ 9.1 µg/mL) among the compounds tested. Carbendazim (IC_50_ 6.3 µg/mL)	[62]
42	*Bipolaris* sp. MU34	*Gynura hispida* Bangkok, Thailand.	Bipolamide B (**119**)	Bipolamide B (**119**) active against *C. cladosporioides*, *C. cucumerinum*, *S. cerevisiae*, *A. niger* and *R. oryzae*, with MIC values of 16, 32, 32, 64 and 64 µg/mL, respectively	[63]
43	*Alternaria alternata* Tche-153	*Terminalia chebula* Rezt. Suanluang Rama IX Public Park, Bangkok, Thailand.	Altenusin (**120**)	Altenusin (**120**) in combination with each of three azole drugs, ketoconazole, fluconazole or itraconazole at their low sub-inhibitory concentrations exhibited potent synergistic activity against *C. albicans* with the FIC index range of 0.078 to 0.188	[64]
44	*Alternaria* sp. UFMGCB 55,	Leaves of *Trixis vauthieri* DC (Asteraceae).	Altenusin (**120**)	The altenusin (**120**) exhibited strong activity against 11 strains *P. brasiliensis* with MIC values ranging between 1.9 and 31.2 µg/mL MIC values found for amphotericin B were between 0.031 and 0.12 µg/mL. Additionally, *S. pombe* cells treated with altenusin were more rounded in shape than untreated cellssuggeststhat altenusin could act through the inhibition of cell wall synthesis or assembly in *P. brasiliensis* and *S. pombe*	[65]
45	*Cladosporium cladosporioides*		Cladosporin (**121**), Isocladosporin (**122**)	At 30 µM compound **121** exhibited 92.7, 90.1, 95.4, and 79.9% growth inhibition against *C. acutatum*, *C. fragariae*, *C. gloeosporioides* and *P. viticola* respectively. Compound **122** showed 50.4, 60.2, and 83.0% growth inhibition at 30 µM against *C. fragariae*, *C. gloeosporioides*, and *P. viticola*, respectively	[66]
46	*Epicoccum* sp. CAFTBO,	*Theobroma cacao* (Sterculiaceae) Mount Kala, near Yaoundé, Centre Province, Republic of Cameroon	Epicolactone (**123**), Epicoccolide A (**124**) and B (**125**)	Compounds **123**–**125** showed inhibitory effects on the mycelial growth of *P. ultimum* and *A. cochlioides* and *R. solani* (MIC in the range of 20–80 µg per paper disc)	[67]
47	*Biscogniauxiamediterranea* Ohu 19B	*Opuntia humifusa* (*Cactaceae*) from the United States	5-methylmellein (**100**)	Compound **100** 5-methylmellein was evaluated for antifungal activity against seven plant pathogens (*C. acutatum*, *C. fragariae*, *C. gloeosporioides*, *F. oxysporum*, *B. cinerea*, *P. obscurans*, and *P. viticola*) using an in vitro microdilution broth assay.	[68]
48	*Emericella* sp. XL029	Leaves of *Panax notoginseng* Shijiazhuang, Hebei Province, China.	5-(undeca-3′,5′,7′-trien-1′-yl)furan-2-ol (**126**) and 5-(undeca-3′,5′,7′-trien-1′-yl)furan-2-carbonate (**127**)	Compound **126** active against *R. solani*, *V. dahliae*, *H. maydis*, *F. oxysporum*, *F. tricinctum*, *B. dothidea*, *and A. fragriae* (MIC values from 25 to 3.1 µg/mL), while compound **127** displayed activity against *V. dahliae*, *H. maydis*, *F. tricinctum*, *B. dothidea*, *and A. fragriae* (MIC values from 50 to 12.5 µg/mL)	[69]
	**Comounds Produced by Hyphomycetes**				
49	*Fusarium fujikuroi* (WF5), *Penicilium chrysogenum* WF6, and *P. expensum* WF7	Finger millet Plants Arkell Field Station, Arkell, ON, Canada.	5-hydroxy 2(3H)-benzofuranone (**128**), dehydrocostus lactone (**129**) and harpagoside (**130**)	Compounds **128**–**130** active against *F. graminearum* with MIC of 31.25, 250.00 and 31.25 µg/mL, respectively.	[70]
50	*Trichoderma koningiopsis* YIM PH30002	*Panax notoginseng*. Wenshan, Yunnan Province, China.	Koninginin O (**131**), koninginin Q (**132**), 7-*O*-methylkoninginin D (**133**)	Compounds **131**–**132** active against *F. oxysporum and P. cucumerina*, with an MIC of 128 µg/mL. Compound **133** showed activity against *P. cucumerina* with an MIC of 128 µg/mL. Nystatin was active with MICs at 32 µg/mL	[71]
51	*Trichoderma koningiopsis* YIM PH30002	*Panax notoginseng*. Wenshan, Yunnan Province, China.	Koningiopisin C (**134**)	Compound **134** exhibited in vitro antifungal activity against *F. oxysporum*, *A. panax*, *F. solani* and *P. cucumerina* with MICs at 32, 64, 32, and 16 µg/mL, respectively	[72]
52	*Trichoderma* sp. 09	*Myoporum bontioides*	Dichlorodiaportinolide (**135**), dichlorodiaportin (**136**)	Compounds **135**–**136** active against *C. musae* and *Rhizoctoniasolani* (MIC values from 6.25 to 150 µg/mL)	[73]
53	*Trichoderma brevicompactum* 0248	*Allium sativum*	Trichodermin (**137**)	Compound **137** active against *R. solani*, *B. cinereal*, *C. lindemuthianum* with an EC_50_ of 0.25, 2.02 and 25.60 µg/mL respectively. Carbendazim showed, antifungal activity against *R. solani*, *B. cinereal*, with an EC_50_ of 0.36 and 10.35 µg/mL respectively	[74]
54	*Trichoderma koningiopsis* YIM PH30002	Wenshan, Yunnan Province of China.	Koninginin R (**138**) and S (**139**)	Compound **138** active against *F. oxysporum* and *F. flocciferum* with MICs at 128 µg/mL, while compound **139** displayed activity against *F. oxysporum* with MIC at 128 µg/mL	[75]
55	*Aspergillus terreus*	*Carthamus lanatus* Al-Azhar University campus, Assiut Branch, Assiut, Egypt.	(22E,24R)-stigmasta-5,7,22-trien-3-β-ol (**140**), aspernolides F (**141**)	Compound **140** active against *C. neoformans* with IC_50_ values of 4.38 µg/mL, amphotericin B (IC_50_ 0.34 µg/mL). Compound **141** showed good activity against *C. neoformans* (IC_50_ 5.19 µg/mL).	[76]
56	*Aspergillus* sp. KJ-9,	*Melia azedarach* which was collected at Yangling, Shaanxi Province, China	Fonsecinone A (**142**), (R)-3-hydroxybutanonitrile (**143**)	Compounds **142** and **143** were active against *G. saubinetti*, *M. grisea*, *B. cinerea*, *C. gloeosporioides* and *A. solani* with MIC range of 6.25–50 µM	[77]
57	*Aspergillus* sp.	*Gloriosa superba* Tirupati, India.	6-methyl-1,2,3-trihydroxy-7,8-cyclohepta-9,12-diene-11-one-5,6,7,8-tetralene-7-acetamide (KL-4) (**144**)	KL-4 (**144**) active against *S. cerevisiae*, *C. albicans* and *C. gastricus* with MIC 25, 12.5, and 50 µg/mL respectively	[78]
58	*Penicillium* sp. R22	*Nerium indicum* collected from Qinling Mountain, Shaanxi Province, China.	5-hydroxy-8-methoxy-4-phenylisoquinolin-1(2H)-one (**145**), 3-*O*-methylviridicatin (**146**) and viridicatol (**147**)	Compound **145** active against *A. brassicae*, *A. alternata and V. mali* with MIC value of 31.2 µg/mL, compound **146** against *A. brassicae*, *B. cinerea* and *V. male* with MIC value of 31.2 µg/mL, compound **147** against *A. brassicae*, *A. alternata* and *B. cinerea* with MIC value of 31.2 µg/mL	[79]
59	*Penicillium raciborskii*,	*Rhododendron tomentosum* were collected at the test site of University of Oulu, Finland.	Outovirin C (**148**)	Outovirin C (**148**) inhibited growth of *F. oxysporum*, *B. cinerea*, and *V. dahlia* at the concentration of 0.38 µM. Compound **148** active against *B. cinerea* (57% inhibition) and slightly less effective against *V. dahliae* (45% inhibition)	[80]
60	*Fusarium* sp.	*Mentha longifolia* Saudi Arabia.	Fusaripeptide A (**149**)	Compound **149** active against *C. albicans*, *C. glabrata*, *C. krusei*, and *A. fumigates* with IC_50_ values of 0.11, 0.24, 0.19, and 0.14 µM, respectively. Amphotericin B exhibited antifungal activity toward *C. albicans*, *C. glabrata*, *C. krusei*, and *A. fumigates* with IC_50_ values of 0.3, 0.6, 0.5, 0.7 µM, respectively	[81]
61	*Fusarium chlamydosporium*	*Anvillea garcinii* Al Madinah Al Munawwarah, Saudi Arabia.	Fusarithioamide A (**150**)	Compound **150** active with inhibition zone diameters 16.2 mm and MIC 2.6 µg/mL towards *C. albicans.* Clotrimazole (inhibition zone diameters 18.5 mm and MIC 3.7 µg/mL)	[82]
62	*Fusarium* sp.	*Ficus carica* Qinling Mountain, Shaanxi Province, China	Helvolic acid Methyl ester (**151**), helvolic acid (**152**) and hydrohelvolic acid (**153**)	Compounds **151**–**153** active against *B. cinerea*, *C. gloeosporioides*, *F. oxysporum* f. sp. *niveum*, *F. graminearum* and *P. capsici* (MIC in the range of 12.5–25 µg/mL), Carbendazim (MIC in the range of 32.2–62.5 µg/mL)	[83]
63	*Fusarium* sp.		Colletorin B (**154**), colletochlorin B (**155**), LL-Z1272β (llicicolin B) (**156**) and 4,5-dihydrodechloroascochlorin (**157**)	Compounds **154**–**156** showed antifungal against *U. violacea* and *F. oxysporum*. Compound **157** showed antifungal activity towards *E. repens*,	Hussain et al. [84]
64	*Curvularia* sp., strain M12,	*Murraya koenigii* Rajshahi University, Bangladesh	Murranolide A (**158**), murranopyrone (**159**), Curvularin (**160**), (S)-dehydrocurvularin (**161**), pyrenolide A (**162**), modiolide A (**163**), and 8-hydroxy-6-methoxy-3-methylisocoumarin (**164**)	Pyrenolide A (**162**) showed a strong motility impairing activity against *Phytophthora capsici* zoospores at a low concentration (100% at 0.5 µg/mL) in a short time (30 min). Compounds **158**–**161** and **163**–**164** exhibited zoospore motility impairment activity at higher concentrations (IC_50_: 50–100 µg/mL)	[85]
65	*Phaeoacremonium* sp.,	*Senna spectabilis* AraraquaraCerrado area, Araraquara, Sao Paulo state, Brazil.	Isoaigialone B (**165**), and C (**166**), aigialone (**167**)	Compounds **165** and **167** exhibited antifungal activity, with a detection limit of 5 µg, for *C. cladosporioides* and *C. sphaerospermum*, compound **166** exhibited weak activity (detection limit > 5 µg), with a detection limit of 25 µg. Nystatin, positive control, showing a detection limit of 1 µg	[86]
66	*Trichothecium* sp.	*Phyllanthus amarus* Pune India.	Trichothecinol A (**168**)	Compound **168** active against *C. albidus* up to 20 µg/mL	[87]
67	*Trichothecium* sp.	*Phyllanthus* sp. Pune India.	Trichothecin (**169**)	Trichothecin (**169**) active against *S. cerevisiae*, *C. albidus var diffluens* (NCIM 3371), *C. albidus* var *diffluens* (NCIM 3372), *F. oxysporum*, *P. expansum*, *T. viride*, *P. varioti* and *A. niger* with MIC of 6.0, 20.0, 12.0, 10.0, 30.0, 40.0, 20.0 and 12.0 µg/mL respectively	[88]
	**Comounds Produced by Basidiomycetes**				
68	*Scleroderma* UFSM Sc1(Persoon) Fries	*Eucalyptus grandis*.	Sclerodol A (**170**) and B (**171**) and related lanostane triterpenoid (**172**)	Compound **170** active against *C. albicans*, *C. tropicalis*, *C. crusei*, *C. parapsiosis* (MIC of 25.0, 25.0, 6.25 and 12.5 MFC 25.0 25.0, 12.5, 25.0 µg/mL) Compound **170** and **172** were active against tested strain (MIC in range of 12.5–100 µg/mL). Nystatin active against test strains (MIC in the range of 0.77–1.52 µg/mL).	[89]

**Table 2:** Spectrum of VOCs emitted by *Muscodor* species and their anti-fungal activity predominantly against plant pathogenic fungi.

No.	Name of the Endophytic Fungi	Geographic Area of Isolation	Major VOCs Produced	Anti-Fungal Activity	Refs.
1	*Muscodor albus*	Central America (Honduras)	2-methylpropanoic acid; 3-methyl-1-butanol; ethanol; acetic acid (methyl ester)	*Rhizoctonia solani*; *Phytophthora cinnamomi*; *Sclerotinia sclerotiorum*; *Fusarium solani*, *Verticillium dahliae*	[91]
2	*M. vitigenus*	South America (Peru)	naphthalene; caryophyllene; azulene	*R. solani*; *Phoma* sp.; *C. coefficola*	[98]
3	*M. roseus*	Australia	2-butenoic acid (ethyl ester); 1,2,4-tri-methyl-benzene; 2-nonadiene	Antifungal spectrum not reported	[101]
4	*M. yucatensis*	South America (Mexico)	caryophyllene; aromadendrene	*Botrytis cineria*; *R. solani*; *C. coefficola*; *Phoma sp.*	[102]
5	*M. fengyangensis*	China	2-methylpropionic acid; β-phellendrene	*B. cineria*; *Aspergillus clavatus*; *Colletotrichum fragiae*; *Sclerotium rolfsii*	[90]
6	*M. crispans*	South America (Bolivia)	2-methylpropanoic acid; ethanol; ethyl acetate	*B. cineria*; *Curvularia lunata*; *P. cinnamomi*; *S. sclerotiorum*	[103]
7	*M. sutura*	USA (Columbia)	butylated hydroxytoleuene; octacecanoic acid; thujopsene; 2-methylpropanoic acid; naphthalene	*Aspergillus fumigatus*; *Colletotrichum lagenarium*; *B. cineria*; *Cercospora beticola*; *Phytophthora palmivora*; *Fusarium solani*	[104]
8	*M. musae*	Thailand	3-methylbutanol acetate 2-methylpropanoic acid	*Alternaria porri*; *Alternaria solani*; *Colletotrichum gloereosporioides*; *Nigrospora oryzae*	[105]
9	*M. oryzae*	Thailand	3-methylbutan-1-ol; 2-methylpropanoic acid	*A. porri*; *A. solani*; *Aspergillus flavus*; *B. cineria*; *C. gloereosporioides*; *N. oryzae*	[105]
10	*M. suthepensis*	Thailand	3-methylpropanoic acid 3-methylbutan-1-ol	*A. porri*; *Alternaria alternata*; *Aspergillus flavus*; *B. cineria*; *C. gloereosporioides*; *Fusarium oxysporum*; *Fusarium solani*; *N. oryzae*	[105]
11	*M. equiseti*	Thailand	3-methylbutan-1-ol; 3-methylbutanoyl acetate; 2-methylpropanoic acid	*A. porri*; *A. solani*; *B. cineria*; *C. gloereosporioides*; *F. oxysporum*; *F. solani*; *N. oryzae*	[105]
12	*M. cinnamomi*	Thailand	2-methylpropanoic acid;2-methyl butanoic acid; azulene	*Rhizoctonia solani*	[105]
13	*M. kashayum*	India	1-methyl-4(1-methylethhylidene)-cyclohexane; 2(4-morpholinyl)ethylamine; 9-octadecanoic acid (methyl ester); 4-octadecylmorpholine	*Bionectria ochroleuca*; *Cercospora beticola*; *Chaetomium heterosporum*; *C. gloereosporioides*; *F. oxysporum*; *Fusarium equiseti*; *Curvularia lunata*	[106]
14	*M. darjeelingensis*	India	4-octadecylmorpholine; 2,6-*bis*(1,1-dimethylethyl)-4-(1-oxopropyl)phenol; beta-aminoethyl-morpholine	*Lasiodiplodia theobromae*; *A. alternata*; *Rhizoctonia solani*; *Cercospora beticola*	[107]
15	*M. strobelii*	India	4-octadecylmorpholine; tetraoxapropellan; aspidofractanine-3-methanol;viridiflorol	*Rhizoctonia solani*; *Colletotrichum gloereosporioides*; *Fusarium oxysporum*; *Lasiodiplodia theobromae*	[108]
16	*M. tigrii*	India	4-octadecylmorpholine; 1-tetradecamine n, n-dimethyl 1,2-benzidicarboxylic acid mono(2-ethylhexyl)ester	*Alternaria alternate* *Cercospora beticola*	[109]
17	*M. heavae*	Thailand	2-phenylethanol; azulene	*Aspergillus niger*; *Phellinus noxius*; *Rigidoporus microporus*	[110]
18	*M. ghoomensis*	India	n,n-dimethyl-1-nonadecamine; 4-octadecylmorpholine	*Cercospora beticola*	[111]
19	*M. indica*	India	n, n-dimethyl-1-pentadecamine; 4-morpholinethanamine	*Cercospora beticola* *Penicillium marnaeffi*	[111]
20	*M. camphora*	India	tetracontane; 4-octadecylmorpholine; n, n-dimethyl-1-pentadecamine	*Colletotrichum gloereosporioides*; *Lasiodiplodia theobromae*	[112]
